# Iron uptake pathway of *Escherichia coli* as an entry route for peptide nucleic acids conjugated with a siderophore mimic

**DOI:** 10.3389/fmicb.2024.1331021

**Published:** 2024-01-31

**Authors:** Uladzislava Tsylents, Michał Burmistrz, Monika Wojciechowska, Jan Stępień, Piotr Maj, Joanna Trylska

**Affiliations:** Centre of New Technologies, University of Warsaw, Warsaw, Poland

**Keywords:** peptide nucleic acid (PNA), siderophores, iron coordination, TonB-dependent transport system, *E. coli* outer-membrane receptors

## Abstract

Bacteria secrete various iron-chelators (siderophores), which scavenge Fe^3+^ from the environment, bind it with high affinity, and retrieve it inside the cell. After the Fe^3+^ uptake, bacteria extract the soluble iron(II) from the siderophore. Ferric siderophores are transported inside the cell via the TonB-dependent receptor system. Importantly, siderophore uptake paths have been also used by sideromycins, natural antibiotics. Our goal is to hijack the transport system for hydroxamate-type siderophores to deliver peptide nucleic acid oligomers into *Escherichia coli* cells. As siderophore mimics we designed and synthesized linear and cyclic N^δ^-acetyl-N^δ^-hydroxy-l-ornithine based peptides. Using circular dichroism spectroscopy, we found that iron(III) is coordinated by the linear trimer with hydroxamate groups but not by the cyclic peptide. The internal flexibility of the linear siderophore oxygen atoms and their interactions with Fe^3+^ were confirmed by all-atom molecular dynamics simulations. Using flow cytometry we found that the designed hydroxamate trimer transports PNA oligomers inside the *E. coli* cells. Growth recovery assays on various *E. coli* mutants suggest the pathway of this transport through the FhuE outer-membrane receptor, which is responsible for the uptake of the natural iron chelator, ferric-coprogen. This pathway also involves the FhuD periplasmic binding protein. Docking of the siderophores to the FhuE and FhuD receptor structures showed that binding of the hydroxamate trimer is energetically favorable corroborating the experimentally suggested uptake path. Therefore, this siderophore mimic, as well as its conjugate with PNA, is most probably internalized through the hydroxamate pathway.

## 1 Introduction

Iron is one of the essential nutrients necessary for bacterial growth, but in the environment, it occurs almost exclusively in an insoluble ferric form (Raymond et al., 2003; Schauer et al., 2008). Therefore, despite its overall abundance, the concentration of the obtainable ferric iron is extremely low (10^−18^ M). The iron access for pathogenic bacteria is restricted even further in the organism of the host (Drakesmith and Prentice, 2012; Johnson and Wessling-Resnick, 2012; Cassat and Skaar, 2013; Wilson et al., 2016; Neumann et al., 2017). In the human body, ferric iron is scarcely accessible (10^−24^ M) since it is either kept intracellularly within hemoglobin in erythrocytes or is extracellularly bound by transferrin or lactoferrin. Iron can be also chelated with lower affinity by other molecules such as albumin, citrate, or even amino acids (Drakesmith and Prentice, 2012; Cassat and Skaar, 2013). Additionally, during the infection, hypoferremic defense response, including hepcidin production by the liver, is activated (Drakesmith and Prentice, 2012; Cassat and Skaar, 2013). Hepcidin is a hormone responsible for the regulation of iron transfer by prohibiting iron cellular efflux and decreasing its extracellular concentration (Drakesmith and Prentice, 2012; Johnson and Wessling-Resnick, 2012; Cassat and Skaar, 2013). To surpass all these obstacles and meet physiological requirements for growth (10^−6^–10^−3^ M of iron; Gumienna-Kontecka and Carver, 2019), bacteria developed several ways to compete for and obtain iron(III), including the production of siderophores (Cassat and Skaar, 2013; Neumann et al., 2017; Kim et al., 2021). Once secreted by the bacterial cell, siderophores capture the ferric iron from the natural environment but also compete for iron with other chelating compounds present in the infected host.

**Figure 1:**
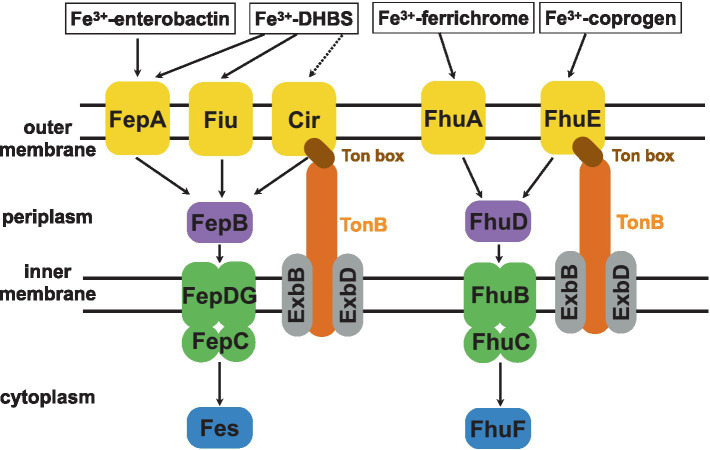
A scheme of the TonB-dependent *E. coli* intake systems of catecholate (enterobactin, 2,3-dihydroxybenzoylserine—DHBS) and hydroxamate (ferrichrome, coprogen) siderophores (Andrews et al., 2003; Braun, 2003). Upon recognition by its specific TBDT, the ferric-siderophore is internalized into the periplasm. This is possible due to the TonB-ExbB-ExbD complex which transmits the energy to the Ton box of appropriate TBDT. This energy is generated by the proton-motive force across the inner membrane. Next, the siderophore binds its respective periplasmic binding protein (catecholate type siderophore - FepB and hydroxamate type - FhuD), which delivers it to the dedicated ABC transporter complex (FepCDG for catecholate and FhuBC for hydroxamate type siderophores) for transport across the inner membrane. Once in the cytoplasm, a dedicated reductase (Fes or FhuF) ‘unpacks’ the Fe^3+^ iron by reducing it to Fe^2+^ for which siderophores show lower affinity.

To uptake iron(III) chelated by siderophores, gram-negative bacteria possess a selective TonB-dependent transporter (TBDT) embedded in the outer membrane and a transmembrane complex containing the TonB, ExbD, and ExbB proteins (Schauer et al., 2008; Noinaj et al., 2010; Delepelaire, 2019; Figure 1). Iron chelators complexed with ferric iron bind to TBDTs with extremely high affinity, exhibiting nanomolar or sub-nanomolar equilibrium dissociation constants (K_d_; Schauer et al., 2008; Josts et al., 2019). Typically, to capture and uptake iron bacteria synthesize a strain-specific siderophore. However, bacteria can also produce several different TBDTs to capture and use xenosiderophores secreted by other species (Delepelaire, 2019; Kim et al., 2021). For example, *E. coli* K-12 synthesizes enterobactin or enterochelin siderophores. Both molecules are recognized by their FepA outer-membrane receptor. However, *E. coli* possesses also other outer-membrane receptors to recognize and uptake Fe(III)-siderophores, such as FhuA (for ferrichrome) and FhuE (for rhodotorulic acid/coprogen; Delepelaire, 2019).

The binding of the siderophore to its specific outer-membrane receptor results in the unfolding of the Ton box, which otherwise remains sequestered (Figure 1; Noinaj et al., 2010). The exposure of the Ton box triggers the interaction between the TBDT and the TonB-ExbB-ExbD complex, which provides energy generated from the proton motive force (Noinaj et al., 2010). This energy is needed for the structural changes of the globular luminal domain (also termed a ‘plug’) required for the transport of the iron chelator across the outer membrane through the TBDT (Schauer et al., 2008). Next, to pass the inner membrane, siderophores are captured by a periplasmic binding protein. Then they are delivered to the inner-membrane-bound ATP-binding cassette of the ABC transporter. The latter transports them further to the cytoplasm, where appropriate reductases convert iron(III) into iron(II), which is released from the siderophore (Schauer et al., 2008; Delepelaire, 2019).

Although iron is one of the key nutrients, its intracellular level has to be controlled to avoid iron accumulation and further oxidation. In *E. coli* this role is assigned to a ferric uptake regulator (Fur) that uses Fe^2+^ as a cofactor to bind to DNA sequences named Fur boxes (fur-regulated promoters; Hantke, 2001; Noinaj et al., 2010). These DNA sequences repress the expression of multiple genes involved in iron metabolism, e.g., iron transporters and proteins involved in siderophore biosynthesis (Hantke, 2001; Noinaj et al., 2010). Thus, Fur regulates iron transport by controlling the expression of TBDTs required for ferric siderophores, transcription of the *tonB* gene, and the *exbB-exbD* operon (Althaus et al., 1999; Chen et al., 2007). Moreover, Fur can bind directly to the promoter region of *fepA-entD*, *fecABCDE*, *fhuACDB*, and *cirA* transport genes (Griggs and Konisky, 1989; Hunt et al., 1994; Angerer and Braun, 1998; Chen et al., 2007; Noinaj et al., 2010). The Fur protein contains a helix-turn-helix motif and two metal-binding sites occupied by Zn^2+^ or other divalent cations (Hantke, 2001; Noinaj et al., 2010). However, when the Fe^2+^ ion binds to the metal-binding site of the Fur protein, it promotes conformational changes and allows Fur binding to the respective Fur boxes (Noinaj et al., 2010; Pasqua et al., 2017). Therefore, when iron is limited, the Fur protein has a lower affinity to DNA and cannot prevent transcription of Fur boxes, leading to increased expression of these genes. Importantly, the Fur-deprived mutants lack iron regulation and do not limit the uptake of iron(III).

Peptide nucleic acid (PNA) is a synthetic nucleic acid analog. PNA has the same nucleobases as DNA, but they are connected via a peptide-like backbone consisting of N-(2-aminoethyl)-glycine units (Nielsen et al., 1991). This neutral backbone eliminates electrostatic repulsion and increases PNA affinity toward natural nucleic acids. As a result, PNA oligomers create stable complexes with either DNA or RNA. Importantly, since PNA structure differs from both peptides and nucleic acids, it is not recognized by proteolytic and nucleolytic enzymes and is resistant to both (Wojciechowska et al., 2020). These advantages highlight the PNA potential in gene targeting strategies, both in diagnosis and as antimicrobial agents [reviewed in Saarbach et al. (2019); Wojciechowska et al. (2020); Perera et al. (2021); Tsylents et al. (2023)].

The main drawback seriously limiting PNA applications is its poor membrane permeability. Another factor limiting PNA uptake in gram-negative bacteria is the lipopolysaccharide (LPS) layer, which forms a negatively charged structural barrier protecting the inner leaflet of the outer membrane and preventing passive diffusion of hydrophobic molecules (Good et al., 2000; Bertani and Ruiz, 2018). Therefore, the conjugation of PNA oligomers to carriers that would enable PNA transport across the membrane has been investigated (Lu et al., 2010; Volpi et al., 2021; Patil, 2022; Patil et al., 2022). One strategy is to use cell-penetrating peptides (CPPs), such as (KFF)_3_K, TAT or (RXR)_4_XB (Bendifallah et al., 2006; Barkowsky et al., 2019, 2022; Patil et al., 2019; Wojciechowska et al., 2020; Yavari et al., 2021; Tsai et al., 2023). Unfortunately, CPPs are not universal as their delivery efficiency depends on a bacterial strain (Goltermann et al., 2022) and they may also be hemolytic (Wojciechowska et al., 2020). The other unique candidate tested as a PNA carrier was the self-assembling DNA tetrahedron (Readman et al., 2017). This DNA structure bound and transported a short PNA oligomer inside a bacterial cell, but the transport mechanism requires further research (Readman et al., 2017). Another non-peptidic strategy, proposed by us, is to use the cell’s active transport system by conjugating the PNA oligomer to vitamin B_12_, which is an essential nutrient required for the growth of most bacteria (Równicki et al., 2017, 2019). Indeed, vitamin B_12_ was found to carry PNA into *E. coli* and *S.* Typhimurium cells. Unfortunately, the concentrations of vitamin B_12_ required by these bacteria are significantly smaller than the concentrations of PNA oligomer necessary to block mRNA translation and achieve the antibacterial effect (Równicki et al., 2019; Wojciechowska et al., 2020).

Following the successful use of vitamin B_12_ as the PNA carrier, we turned to microbial iron regulation mechanisms, which also seem viable for the Trojan horse strategy. A similar approach (reviewed by (Negash et al., 2019; Zhanel et al., 2019)), where conjugation of siderophores with various antibiotics was attempted, resulted in several promising β-lactam–siderophore conjugates. Using siderophore mimics should allow us to take advantage of the active transport of a limited yet essential nutrient that is required in amounts similar to the PNA concentrations we would like to deliver. Therefore, we hypothesized that siderophore mimics are promising candidates for PNA carriers through the TonB-dependent transporters. In this work, we designed and synthesized hydroxamate-based iron chelators mimicking the natural ferrichrome and coprogen. We then verified their iron-binding properties, the interactions of iron with the siderophore at an atomistic level of detail, as well as the path of their uptake. Next, we tested the carrier potential of one of these siderophores by conjugating it with the PNA oligomer aimed at silencing the expression of the reporter gene in *E. coli*.

## 2 Results and discussion

### 2.1 Design and synthesis of siderophore mimics

Based on the structures of the ferrichrome and coprogen, well-known hydroxamate-type natural siderophores, recognized by, respectively, the FhuA and FhuE *E. coli* TBDT receptors (Figure 1), we designed two synthetic siderophore mimics – the linear (S_L_) and cyclic (S_C_). To synthesize these siderophores, we used a commercially available modified ornithine (N^α^-Fmoc-N^δ^-acetyl-N^δ^-benzyloxycarbonyl-l-ornithine). This building block is suitable for solid-phase peptide synthesis (SPPS) and provides the N^δ^-terminal hydroxamate groups that are crucial for metal binding. These ornithine derivatives were used to obtain a linear N^δ^-acetyl-N^δ^-hydroxy-l-ornithine trimer (Orn(Ac,OH))_3_ to preserve the same number of metal-binding groups as in ferrichrome and coprogen and to achieve the optimal number of hydroxamate groups for iron coordination (Minnick et al., 1992; Boukhalfa and Crumbliss, 2002).

**Figure 2:**
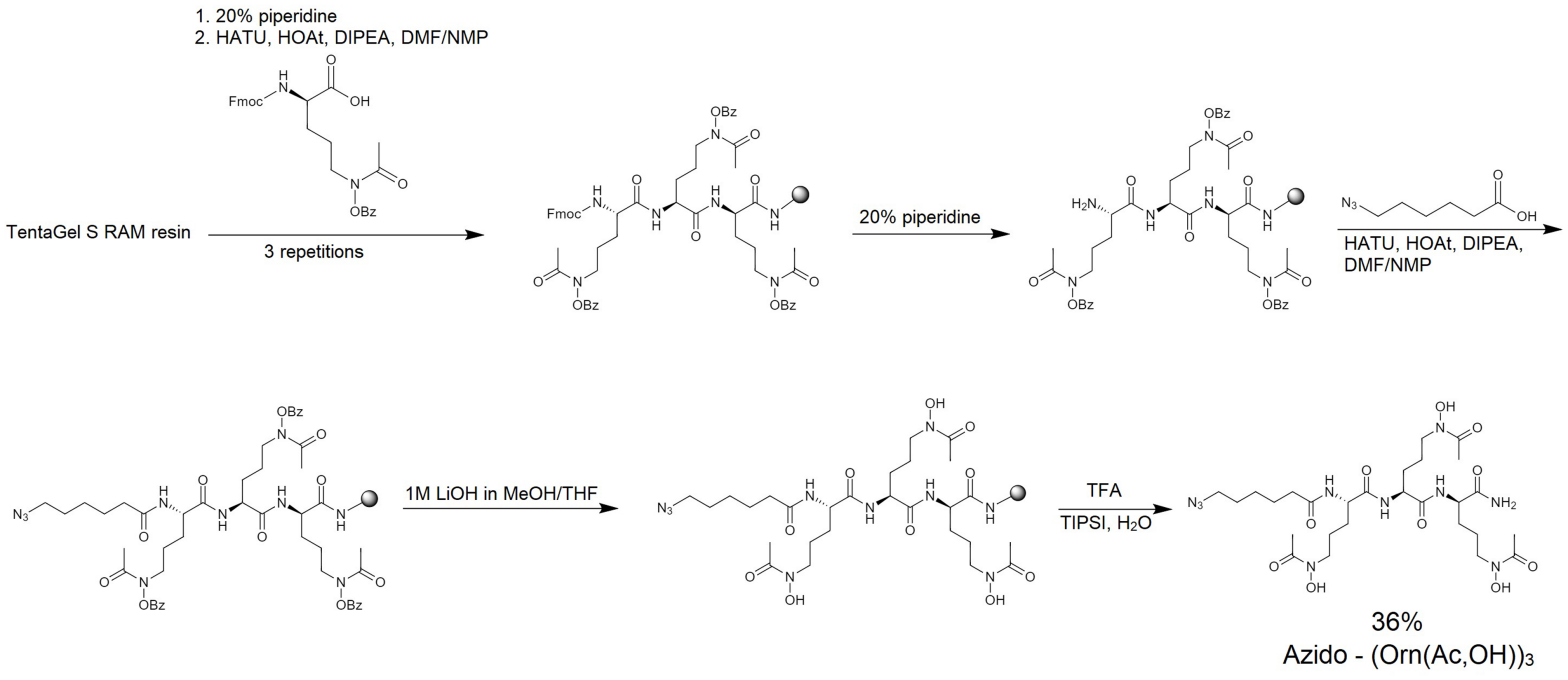
The synthesis scheme of azido-(Orn(Ac,OH))_3_ – named S_L_.

**Figure 3:**
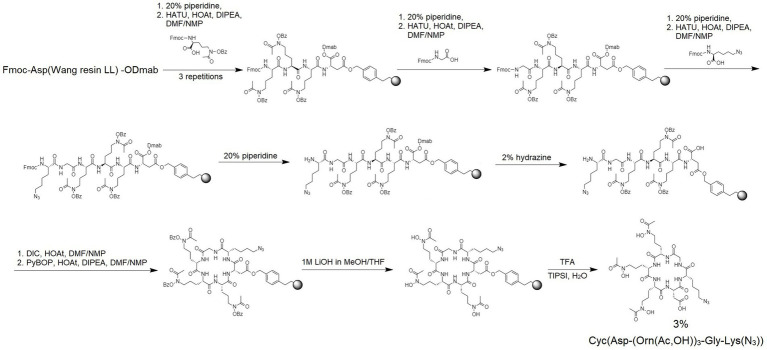
The synthesis scheme of azido-Cyc(Asp-(Orn(Ac‚OH))_3_-GIy-Lys(N_3_)) – named S_C_.

Ferrichrome is a siderophore forming a cyclic structure. Therefore, after the synthesis of the linear siderophore mimic S_L_ (Figure 2), we obtained a cyclic analog using the head-to-tail cyclization strategy (Figure 3). Synthesis details are provided in the Materials and Methods section. Cyclization was attempted to increase the proteolytic stability and to add structural constraints to the peptide backbone. Restricting the flexibility of the hydroxamate groups could decrease their conformational freedom to make them adopt a conformation that would stably coordinate iron(III). Therefore, we proceeded to test the iron coordination properties of both linear and cyclic siderophore mimics.

### 2.2 Iron coordination by siderophore mimics

Upon binding iron, siderophores with three hydroxamate groups, where six oxygen atoms are used for Fe^3+^ coordination, form the octahedral hexacoordinate complex. This specific structure has two optical isomers: Δ (right-hand propeller) and Λ (left-hand propeller), which are separate from geometric isomers (van der Helm et al., 1980). Natural siderophores with chelated iron(III) adopt the structure of one of the most optimal optical isomers. For example, the Fe^3+^-desferrichrome complex adopts the Λ configuration (van der Helm et al., 1980), but for ferric-coprogen, the Δ configuration is optimal although the Λ configuration is also present (Wong et al., 1983; Winkelmann, 1991). Importantly, it was shown that specific receptors responsible for the siderophore transport can recognize optical isomers (Winkelmann, 1979; Thulasiraman et al., 1998).

To elucidate the configuration of the siderophore-iron(III) complexes, we used circular dichroism (CD) spectroscopy where the minimum and maximum with Cotton effect are expected in the range of 350–490 nm (Winkelmann, 1991; Matsumoto et al., 2001). For the Λ configuration, the minimum band in the CD spectrum should be present before the maximum. Since the Δ configuration is a mirror image of the Λ configuration, the maximum band appears first (Winkelmann, 1991). In the presence of the Fe^3+^ salt, the natural siderophores and S_L_ solution changed from colorless to yellow after several minutes. This indicates the presence of the siderophore-ferric iron complex and further confirms the metal-binding properties of these compounds (Winkelmann, 1991). Based on this observation, during the CD experiments, for every siderophore, we adjusted the incubation time at the optimal concentration, ranging from 40 to 90 min (see Supplementary Figure S1).

**Figure 4:**
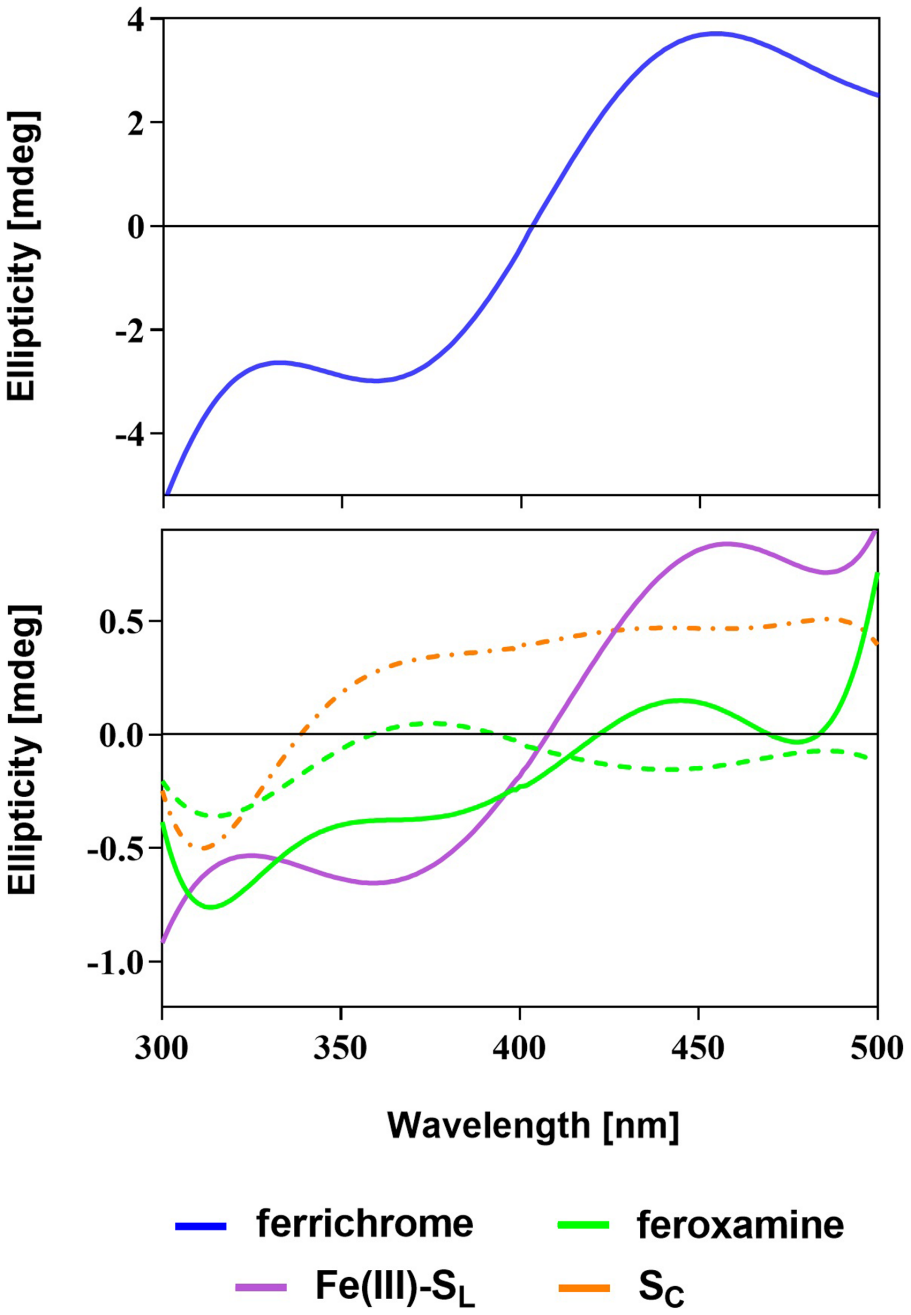
CD spectra of the natural (ferrichrome and feroxamine) and synthetic (S_L_ and S_C_) siderophores with Fe^3+^ salt solution in phosphate buffer (pH = 7.0). Optimal Λ (solid line) or Δ (dashed line) configuration was observed for siderophore-iron(III) complexes apart from S_C_ (dashed-dotted line).

CD spectra show that S_L_ chelates ferric iron adopting the structure of the Λ isomer exhibiting the Cotton effect with the negative (360 nm) and positive (458 nm) bands (Figure 4). Similar positions of the minima and maxima in the Fe(III)-S_L_ complex and ferrichrome spectra suggest that, while capturing iron, S_L_ adopts a structure comparable to the cyclic ferrichrome (Table 1). However, the signal intensity for S_L_ is not as notable as for ferrichrome even though the concentrations are equal. This suggests the presence of the Δ configuration since linear siderophores are flexible (Wong et al., 1983). On the other hand, S_L_ is not as flexible as deferoxamine, which can appear as a racemic mixture of two optical isomers (Hossain et al., 1986). For deferoxamine and its complex with Fe^3+^, known as feroxamine, sometimes the Λ configuration can be assigned, and later the Δ isomer is more prominent, but the signal intensity could not be improved either with increasing concentration or incubation time.

**Table 1:** Summary of the CD spectroscopy experiments.

Siderophore	Wavelength [nm]		Optimal conditions		Optimal Fe^3+^ complex configuration
	Minimum	Maximum	Concentration [mM]	Incubation time [min]	
Ferrichrome	360	456	1.00	60–90	Λ
Feroxamine	371 or 445	371 or 445	0.24	40	Λ and Δ
Fe(III)-S_L_	360	458	1.00	60	Λ
S_C_	–	487	–	–	–

The CD spectrum of S_C_ with Fe^3+^ does not resemble the spectrum of any natural siderophore and does not fit the description of the Δ or Λ isomers. The minimum is observed but the required Cotton effect is not present in the region between 360 and 450 nm. Additionally, no color change was observed for the mixture of S_C_ and Fe^3+^ salt solutions throughout the whole duration of the experiment. This strongly indicates that S_C_ cannot bind ferric iron. This is probably due to the structural hindrance or rigidity of S_C_, which does not allow the proper arrangement of the hydroxamate oxygen atoms for iron coordination. Therefore, based on these findings, further experiments and simulations focused solely on the S_L_ or its conjugates with PNA.

### 2.3 Growth recovery assays confirm S_L_ is a functional siderophore recognized by the FhuE receptor

After confirming that S_L_ binds iron(III), we tested if the S_L_ molecule can be used by *E. coli* as a siderophore. *E. coli* uses several siderophore-mediated iron uptake systems (Andrews et al., 2003). Each of them consists of one or more outer-membrane receptors, periplasmic binding protein, inner-membrane transporter, and cytoplasmic reductase (Figure 1). The two most prominent iron uptake systems recognize catecholate- and hydroxamate-type siderophores. To identify the uptake path of S_L_ and verify its siderophore function as the iron-delivering compound, we performed a growth recovery assay adopted from Zheng et al. (2012). In this assay, bacteria are cultured in conditions that limit their growth, which we ensured by the presence of iron-chelating agent - 2,2′-dipyridyl (DP). Two parallel cultures were prepared, with and without the addition of the S_L_ siderophore mimic. If S_L_ works as a functional siderophore, then we should observe a growth increase in the culture that contains S_L_. If no growth recovery or increase is observed, then either the siderophore is non-functional or its use is prohibited, e.g., by the lack of a protein in the uptake system. Thus, we tested various *E. coli* K-12 mutants in which the genes coding for proteins of these transport systems were deleted (Table 2).

**Table 2:** Bacterial strains used in this study.

Strain (deleted gene)	Description
*E. coli* K-12 MG1655	Wild-type *Escherichia coli* laboratory strain
*E. coli* JW2142-1 (*cir*)	Unable to capture products of enterobactin hydrolysis
*E. coli* JW0790-2 (*fiu*)	Unable to capture products of enterobactin hydrolysis
*E. coli* JW1088-5 (*fhuE*)	Missing the outer-membrane coprogen receptor FhuE
*E. coli* JW0576-2 (*fes*)	Missing the Fes enzyme responsible for Fe(II) release from enterobactin
*E. coli* JW5086-3 (*fepA*)	Missing the outer-membrane enterobactin receptor FepA
*E. coli* JW0584-1 (*fepB*)	Missing the periplasmatic protein FepB involved in the transport of enterobactin and DHBS
*E. coli* JW0669-2 (*fur*)	Missing Fur - transcriptional repressor of genes responsible for siderophore receptor production
*E. coli* JW0146-2 (*fhuA*)	Missing the outer-membrane ferrichrome receptor FhuA
*E. coli* JW0148-1 (*fhuD*)	Missing the periplasmatic protein FhuD involved in the transport of ferrichrome, ferrioxamine, and coprogen

**Figure 5:**
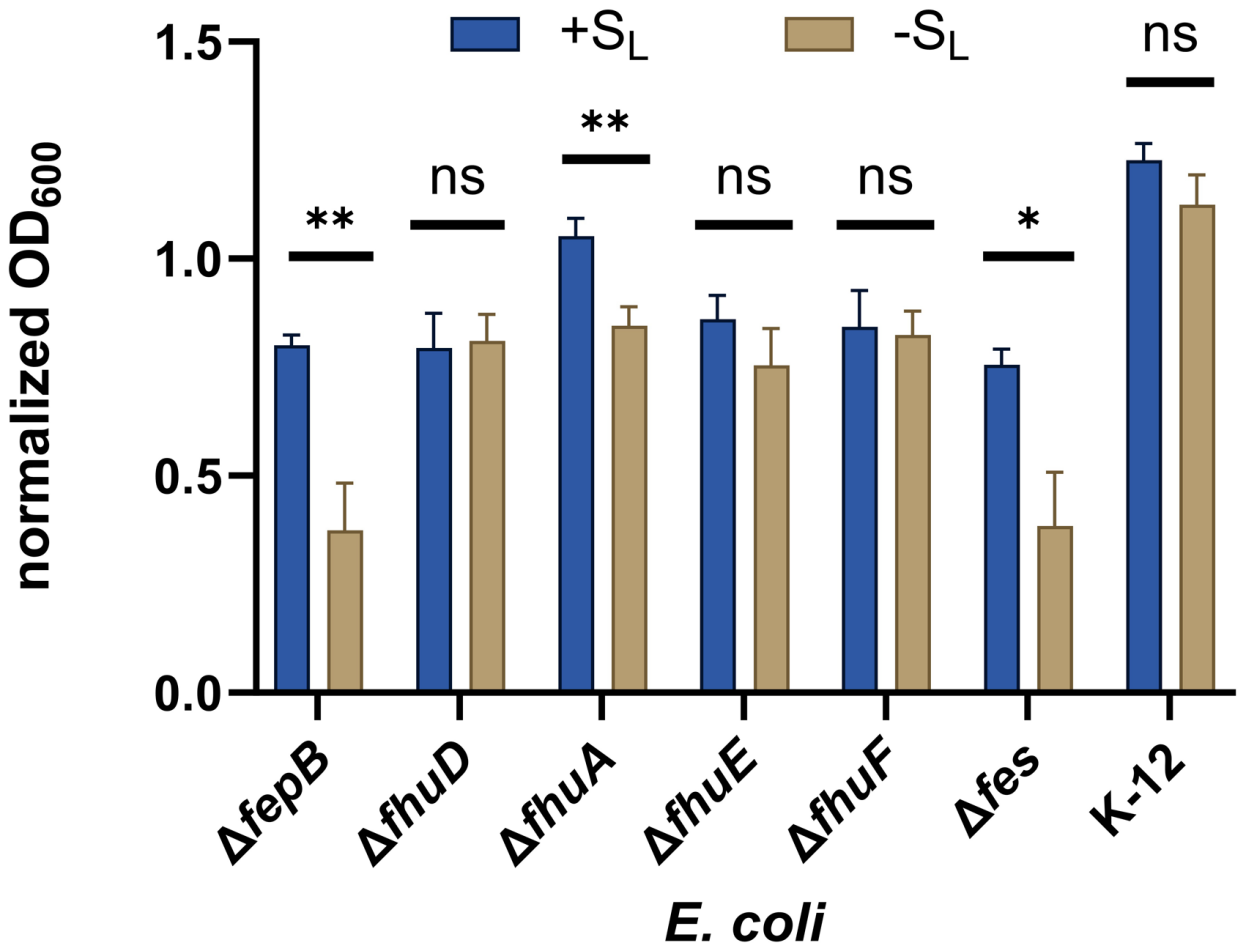
Growth recovery assay. Various *E. coli* mutants were cultured in iron-limiting conditions with or without S_L_ (at a concentration of 16 μM). The growth of different strains was measured by OD_600_ and normalized to OD_600_ measured for a given strain in non-iron limiting conditions. The experiment was repeated three times on different days. The errors shown are SEM, *n* = 4–6. The statistical significance of the difference in growth was verified by a two-tailed t-test (**p < 0.01, *p < 0.05, ns: non-significant).

First, we tested mutants lacking the FepB and FhuD proteins, which are the periplasmic binding proteins in the catecholate- and hydroxamate-type siderophore uptake systems, respectively (Figure 1). Growth recovery was observed for the FepB mutant, whereas no significant change in bacterial growth for the mutant lacking FhuD was detected (Figure 5). These results were further confirmed by testing mutants lacking cytoplasmic siderophore reductases for the catecholate (the Fes protein) or hydroxamate (the FhuF protein) systems. The output indicates that S_L_ is internalized via the hydroxamate siderophore uptake system. This is consistent with our expectations because S_L_ mimics the hydroxamate-type siderophore. Next, we proceeded to identify the outer-membrane receptor for S_L_. Two outer-membrane receptors belong to the hydroxamate uptake system: the ferrichrome receptor (FhuA) and the coprogen receptor (FhuE; Killmann and Braun, 1998; Figure 1). Mutants deficient in these receptors were tested in growth recovery assays showing statistically significant recovery for the FhuA mutant but not for the FhuE mutant (Figure 5). In summary, the growth recovery assays confirmed that S_L_ is a functional siderophore for *E. coli*, and indicated that S_L_ is recognized by the coprogen outer-membrane receptor FhuE and internalized through the hydroxamate pathway.

### 2.4 The S_L_ siderophore mimic transports PNA to *Escherichia coli* cytoplasm

**Figure 6:**
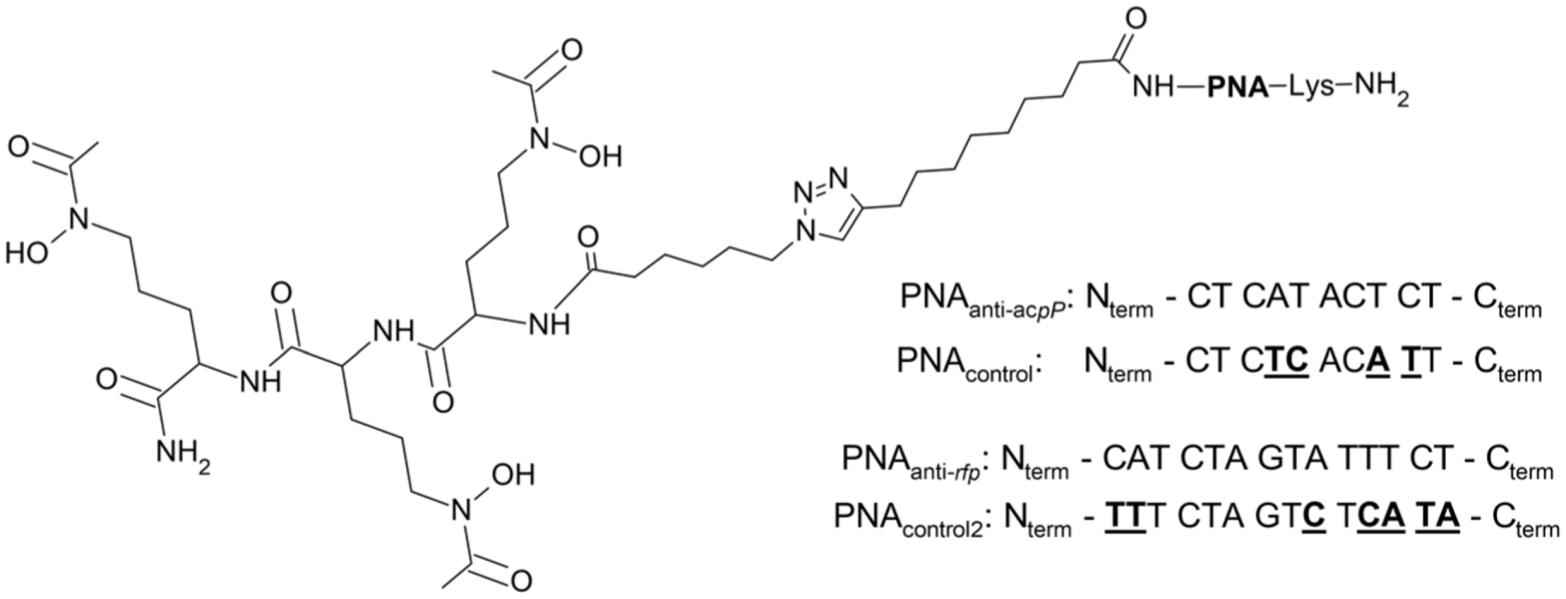
Schematic structure of the S_L_-PNA conjugate with the PNA sequences used. The PNA_anti-*acpP*_ sequence was used to silence the expression of the *acpP* gene encoding the acyl carrier protein and PNA_anti-*rfp*_ to silence the *mrfp1* gene encoding the red fluorescent protein (see text). The control scrambled PNA sequences for PNA_anti-*acpP*_ (PNA_control_) and PNA_anti-*rfp*_ (PNA_control2_) are shown with underlined mismatched bases.

After we confirmed that S_L_ is internalized by *E. coli* K-12, we tested if this siderophore mimic acts as a PNA carrier through the *E. coli* cell wall. For this aim, we conjugated S_L_ with different PNA oligomers. Based on the CD experiments, only S_L_ was used for conjugation. This siderophore mimic was synthesized with a linker possessing an azide moiety needed for its conjugation with PNA. The conjugation was performed using the copper-catalyzed azide-alkyne cycloaddition (CuCAAC) reaction resulting in the non-cleavable linkage via the triazole ring (Figure 6).

**Figure 7:**
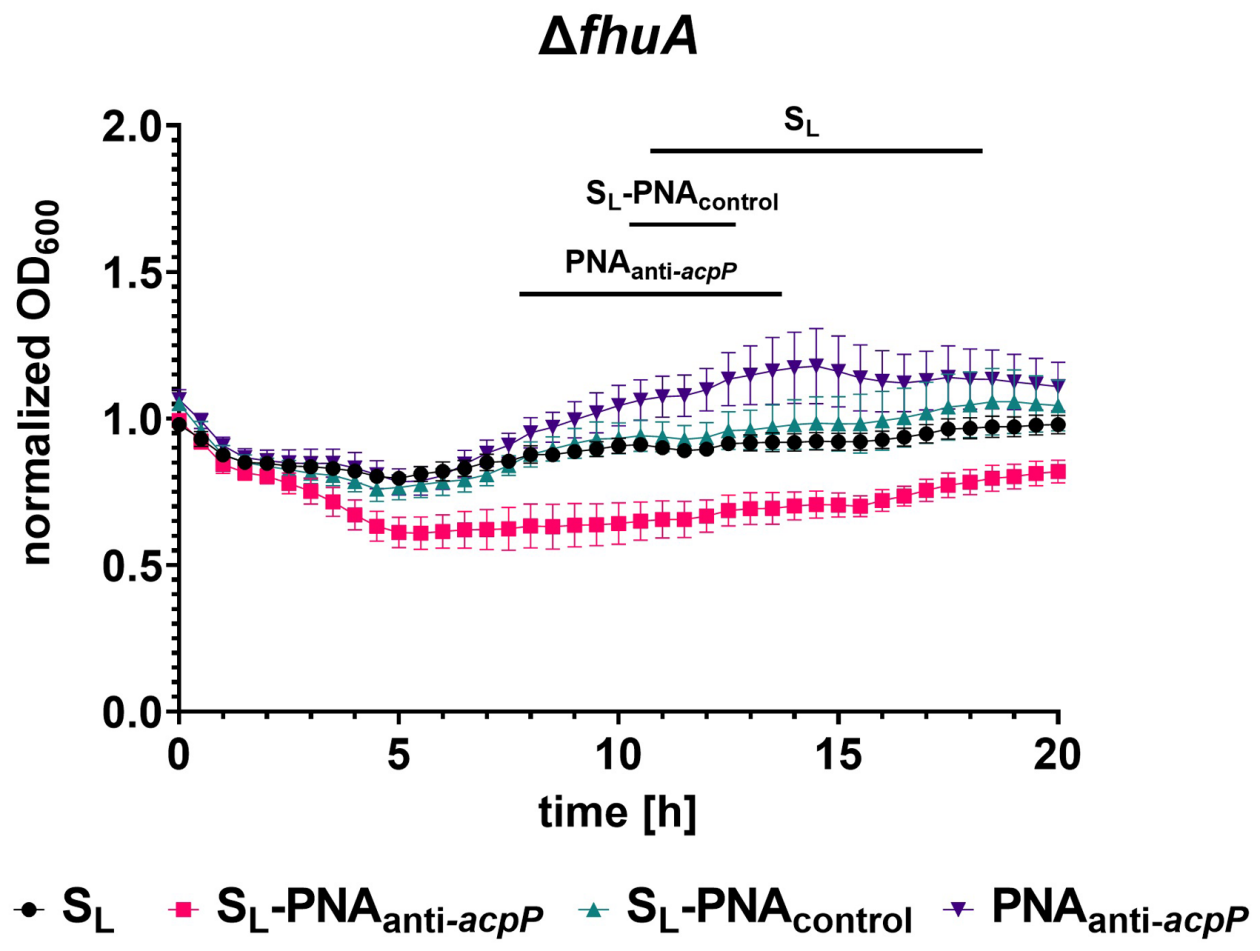
Kinetic measurement of the growth recovery assay for the Δ*fhuA* strain cultured in iron-limiting conditions without or with S_L_ and its conjugate with PNA_anti-*acpP*_ or PNA_control_ (each at a concentration of 16 μM). The growth was normalized to the OD_600_ measured without the addition of any compound. The experiment was performed in two biological replicates, and two technical replicates each. The errors shown are SEM, *n* = 4. Statistical significance was tested by the two-way ANOVA test with horizontal lines showing periods for which a significant difference (*p* < 0.05) was observed between S_L_-PNA_anti-*acpP*_ and other compounds.

To test if S_L_ can transport PNA oligomers to *E. coli* cells, we first performed an altered growth recovery assay in which instead of S_L_ alone we used its conjugate with PNA. The sequence of the PNA was designed to hybridize with mRNA of the *acpP* gene (Figure 6) and as a consequence silence its expression (Good et al., 2001). The *acpP* encodes for acyl carrier protein involved in the fatty acid synthesis pathway and is one of the *E. coli* housekeeping genes (Nejad et al., 2021). We expected that silencing of the *acpP* gene would result in a measurable growth inhibition as observed previously for PNA_anti-_*_acpP_* conjugated to peptide carriers (Good et al., 2001; Hansen et al., 2016; Castillo et al., 2018). To our surprise, the endpoint experiment showed no significant growth differences in bacteria treated with S_L_-PNA_anti-*acpP*_ conjugate (data not shown). To obtain better insight into this phenomenon, we performed a kinetic experiment during which bacterial growth was monitored every 30 min for 20 h of culture (Figure 7; Supplementary Figure S2). To ensure the conjugate was internalized into bacterial cells, we used the *E. coli* K-12 mutants Δ*fepB*, Δ*fhuA*, and Δ*fes* for which we already confirmed the uptake (Figure 5). When treated with S_L_-PNA_anti-*acpP*_, no statistically significant growth differences were observed for the Δ*fepB* and Δ*fes* mutants (Supplementary Figure S2). On the contrary, the Δ*fhuA* strain showed initial growth inhibition followed by later recovery (Figure 7). We suspect that this growth pattern may be the result of initial partial growth inhibition during the early stages of the culture when the ratio of the conjugates to bacterial cells is high. As the bacteria grow in numbers, this ratio drops which reduces the inhibition effect and allows growth recovery. Therefore, this experimental design may carry an internal flaw, namely the inability to separate the growth-promoting effect of the siderophore from the growth-inhibiting effect of PNA_anti-*acpP*_.

To overcome the above limitations of the *acpP*-based assay, we designed another experiment in which we cultured the *E. coli* K-12 strains expressing the red fluorescent protein (RFP) in iron-limiting conditions. We used the protocol based on our previous studies on PNA conjugates with vitamin B_12_ (Równicki et al., 2017; Pieńko et al., 2021), but the culture was supplemented with the conjugate of S_L_ and PNA. To silence the RFP expression, the PNA_anti-*rfp*_ oligomer targeted the mRNA of the *mrfp1* gene encoding RFP (Figure 6). A 14-mer long PNA sequence complementary to the start codon and covering five nucleotides of the ribosome binding site (RBS) in mRNA was used. This approach assumes that if the conjugate of S_L_ and PNA reaches the bacterial cytoplasm, it would bind and sterically block the complementary mRNA fragment. As a result, RFP expression will be reduced, which would manifest itself in a fluorescence decrease. The change in fluorescence should not be affected by the suggested growth promotion effect of the siderophore, so the results obtained for the PNA_anti-*rfp*_ sequence should provide clearer insight into the carrier potential of S_L_. Here, fluorescence was measured using the flow cytometer. We chose this method over the traditional plate-based assay because it enables insight into individual cells. Thus, it is less prone to noise allowing for more precise measurements (Ambriz-Aviña et al., 2014).

**Figure 8:**
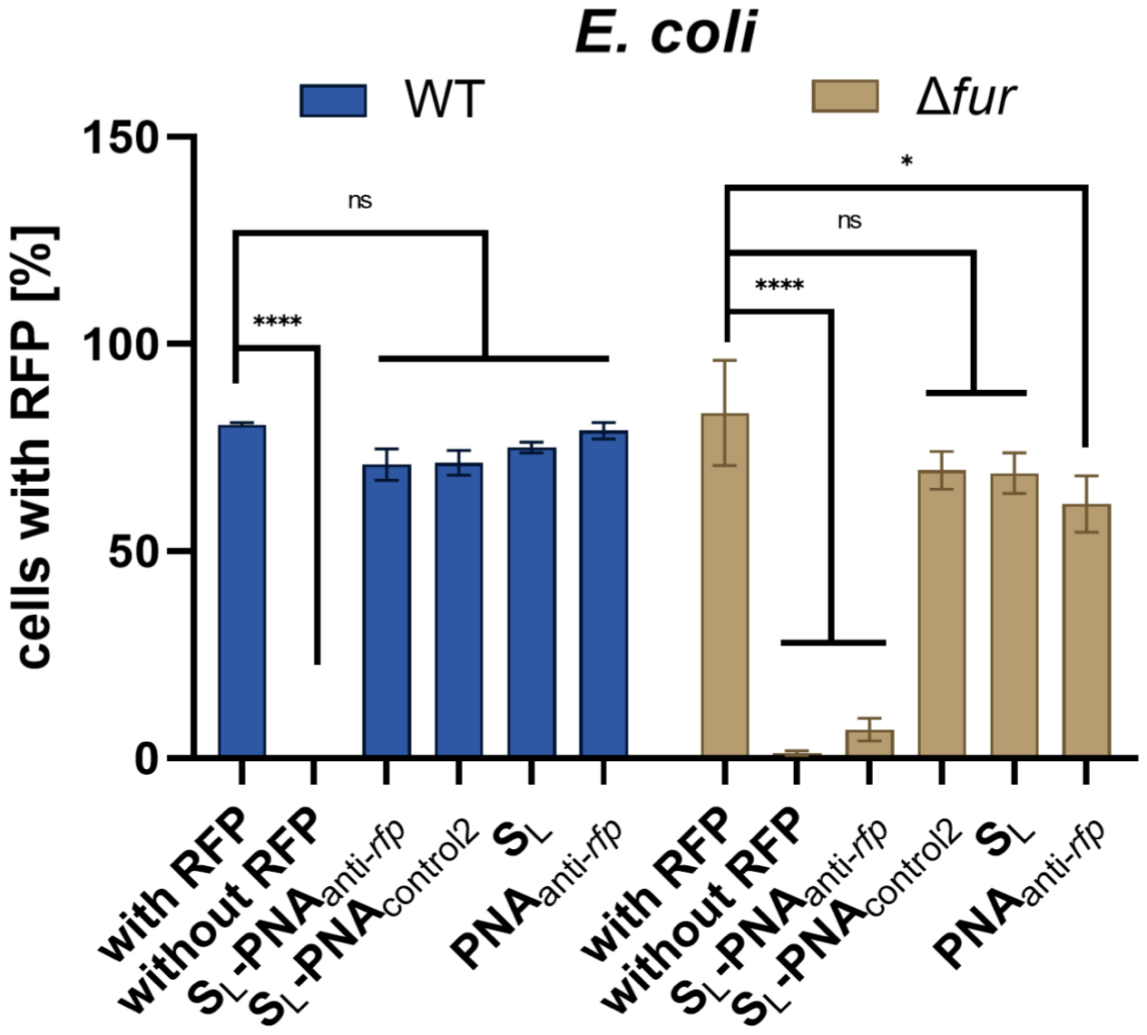
RFP fluorescence silencing in *E. coli* K-12 wild type and *Δfur* mutant. Bacteria were cultured in iron-limiting conditions and with the addition of various compounds (at a concentration of 16 μM). Bacterial cells were tested for RFP fluorescence using the flow cytometer with gates set up based on two control cultures: bacteria expressing RFP and bacteria lacking the RFP gene. The experiment was repeated three times on different days. Errors are SEM, *n* = 3. Statistical significance of the observed differences was determined by a two-way ANOVA test (****p < 0.0001, *p < 0.05, ns, non-significant).

Initially, we tested the wild-type *E. coli* K-12 (Figure 8). As expected, we observed no reduction of RFP fluorescence when the culture was supplemented with S_L_, PNA_anti-*rfp*_, and S_L_-PNA_control2_ (the scrambled PNA sequence not fully complementary to the targeted mRNA transcript; Figure 6). Indeed, as found by us (Wojciechowska et al., 2014, 2020; Równicki et al., 2017) and other groups (Good et al., 2000; Bendifallah et al., 2006; Readman et al., 2017; Barkowsky et al., 2019), PNA itself is not able to penetrate cell membranes without additional help. The scrambled PNA_control2_ sequence in the S_L_-PNA_control2_ conjugate is only partially complementary to the RFP-coding transcript (with seven mismatches) and does not overlap with the start codon or ribosome binding site (RBS), so it served as a negative control.

Surprisingly, we did not observe statistically significant RFP silencing for the fully complementary S_L_-PNA_anti-*rfp*_. We hypothesize that although the S_L_-PNA_anti-*rfp*_ molecule reaches the bacterial cytoplasm and hybridizes with the proper mRNA fragment, it lacks sufficient numbers to saturate enough binding sites to cause a detectable reduction in RFP fluorescence. Since PNA molecules are insensitive to intra-cellular enzymatic degradation it should be possible to increase the number of S_L_-PNA_anti-*rfp*_ per cell by stimulating the siderophore uptake. Bacteria were already cultured in iron-limiting conditions, which should induce such uptake. However, as proved in the growth recovery assays, as S_L_ enters bacterial cells it provides them with iron, which may reduce the further siderophore uptake over time. What is more, *E. coli* can produce enterobactin and acquire iron independently to S_L_, which in turn would reduce intake of S_L_ even further. Thus, we decided to perform this experiment using the *E. coli* K-12 Δ*fur* mutant (Figure 8).

Fur is a global iron-dependent regulator of *E. coli* that controls the expression of more than 90 genes (Escolar et al., 1999). It works as a positive repressor, which means that in a high iron environment, Fur binds to iron and causes repression of genes involved in iron acquisition. If Fur is missing then bacteria keep acquiring iron no matter if its demand was already satisfied. Indeed, the flow cytometry data acquired for *E. coli* K-12 Δ*fur* showed a substantial loss of RFP fluorescence for bacteria cultured in the presence of S_L_-PNA_anti-*rfp*_ (Figure 8). No other compound showed a statistically significant difference to the positive control.

**Figure 9:**
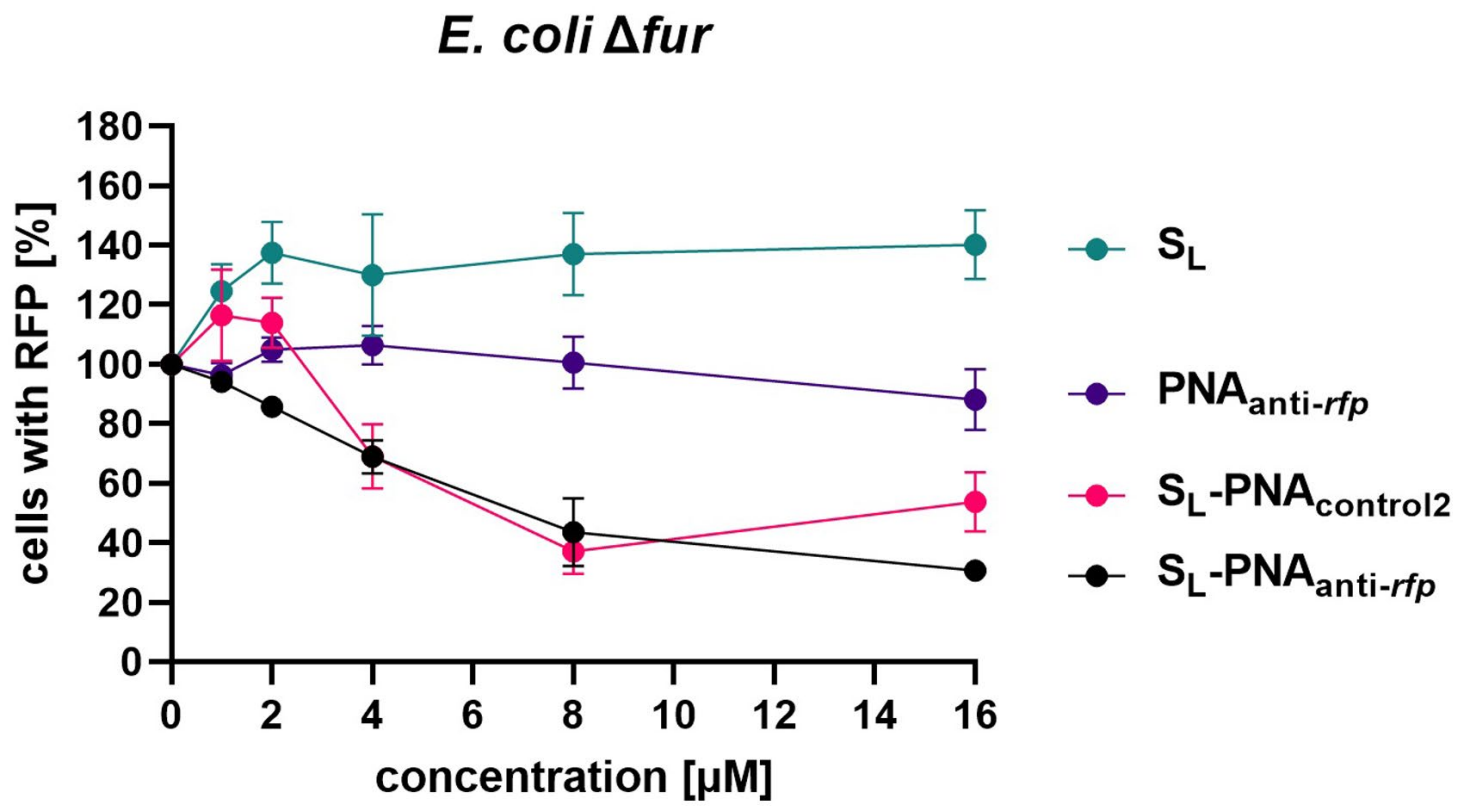
Dose-dependent RFP fluorescence silencing in the *E. coli* K-12 *Δfur* mutant. Bacteria were cultured in iron-limiting conditions and with the addition of various compounds (at concentrations ranging from 0 to 16 μM) preloaded with iron. Bacterial cells were tested for RFP fluorescence using the flow cytometer with gates set up based on two control cultures: bacteria expressing RFP and bacteria lacking the RFP gene. Results were normalized to respective Growth Control values. The experiment was repeated three times on different days. Errors shown are SEM, *n* = 3.

To further verify the results obtained for *E. coli Δfur*, we performed a dose–response experiment (Figure 9). PNA alone did not affect the observed RFP levels at any concentration tested. Unconjugated S_L_ showed an increased RFP cell count, which probably reflects the ‘extra-nutrition’ effect provided by S_L_ acting as a functional siderophore delivering iron. As expected, increasing the concentration of the S_L_-PNA_anti-*rfp*_ conjugate reduced, in a dose-dependent way, the RFP cell count. However, interestingly, S_L_-PNA_control2_ increased the RFP count at low concentrations (up to 2 μM) and decreased at higher concentrations similarly to the S_L_-PNA_anti-*rfp*_. This may be the result of an interplay between two contradictory effects, the first being the siderophore effect, and the second one – the PNA-dependent RFP silencing. Although PNA_control2_ sequence is only half-complementary to the mRNA fragment (with seven mismatched bases, including replacement of one pyrimidine by another in three separate positions) as compared to the PNA sequence, at concentrations of 4 and 8 μM, it seems to silence the RFP expression similarly as the dedicated PNA. However, for higher concentrations (16 μM) it is no longer as efficient. This leaves room for further investigation into the secondary structures and interactions between PNA_anti-*rfp*_ and RNA molecules, as well as improvement of PNA sequence design. Altogether, our results indicate the possibility of using siderophore mimics, such as S_L_, to carry the PNA into the bacterial cells and to modify the expression of a particular gene.

### 2.5 Molecular dynamics of iron(III) binding to the linear N^δ^-acetyl-N^δ^-hydroxy-l-ornithine trimer

Coordination of iron(III) was detected by CD spectroscopy only for the S_L_ siderophore. To understand the dynamics of this process at an atomistic level of detail, especially the flexibility of the siderophore mimic, we performed microsecond-long classical molecular dynamics simulations of various S_L_ variants without and in the presence of the Fe^3+^ ion (see Methods; Supplementary Figure S3).

Simulations of the free S_L_ showed that this modified ornithine trimer in solution is internally flexible and does not adopt any preferential structure. The atomic root-mean-square fluctuations (RMSF) suggest that each S_L_ monomer is equally mobile (see exemplary RMSF in Supplementary Figure S4). Also, the clustering analyses pointed to four clusters with occupancies between 20 and 30% each (Supplementary Figure S5).

The simulations with the iron(III) ion, initially randomly positioned in solvent away from S_L_, reproduced the association and binding of iron(III) to this siderophore. The hydroxamate-type siderophores bind iron(III) via electrostatic attraction coordinating up to six oxygen atoms belonging to the three -NOH-CO- groups. However, binding is connected with the dissociation of protons bound to the oxygen atoms, and with positions of the oxygen atoms of the hydroxamate groups in the *cis* conformation.

**Figure 10:**
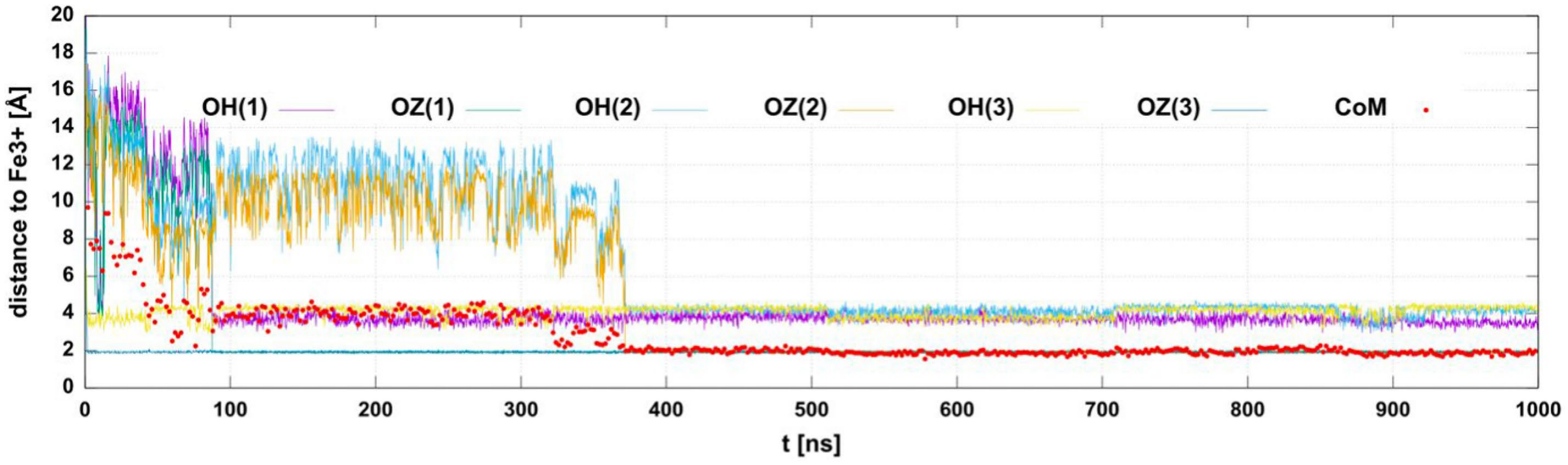
The distances between the Fe^3+^ ion and hydroxamate oxygens or the center of mass (CoM) of the siderophore as a function of the simulation time. The plot shows distances to the six hydroxamate group O-atoms (OH or OZ in the deprotonated *cis* form with the S_L_ residue number in parenthesis). The distance to CoM is marked with red circles. The CoM was calculated for all non-hydrogen atoms.

We monitored the distances of the iron(III) ion to the six oxygen atoms of the three hydroxamate groups. In eight simulations of the siderophore with deprotonated hydroxamate oxygens in the *cis* configuration we observed at least 4 oxygens interacting with iron(III). In two simulations, we found 6 oxygens close to Fe^3+^. The distances of iron(III) to the coordinating oxygens in one of the trajectories are shown in Figure 10, showing that two oxygens position as close as 2 Å to the ion.

**Figure 11:**
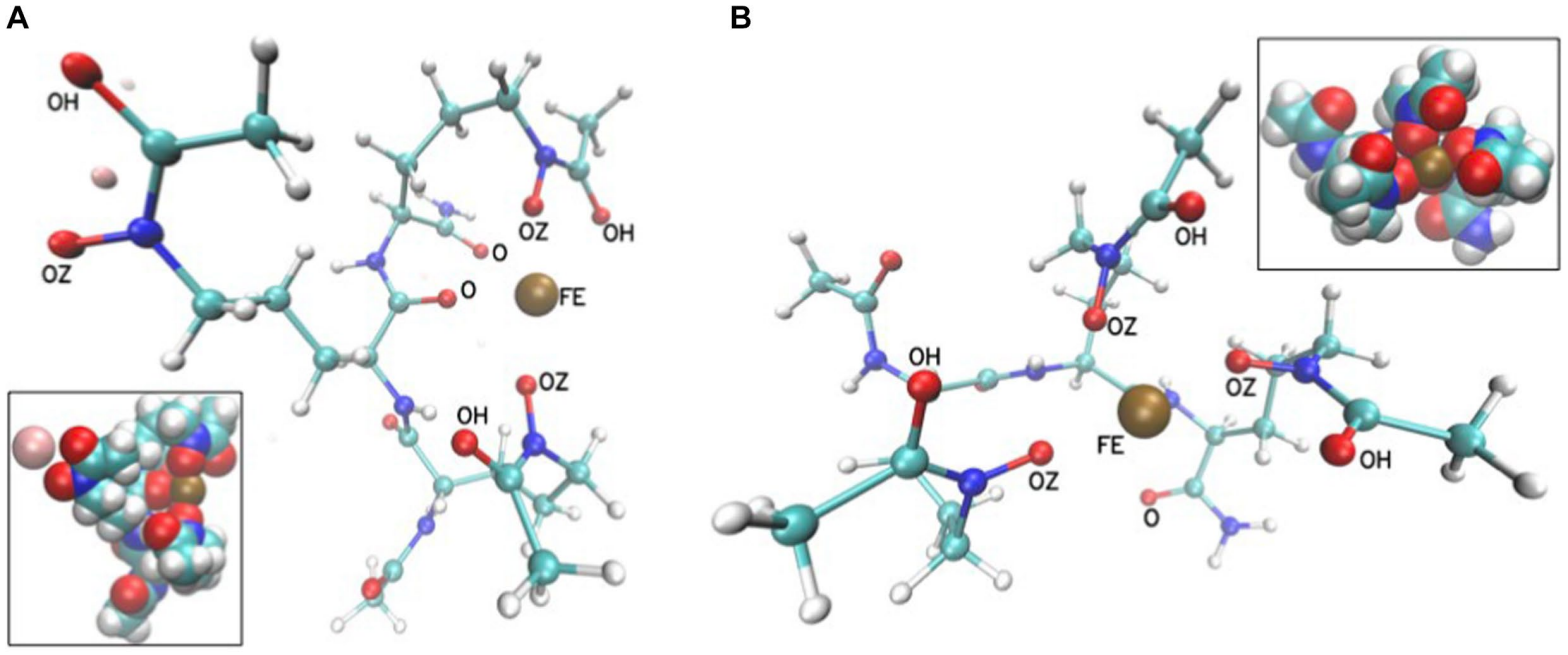
Exemplary conformations in a ball and stick representation from MD simulations of S_L_ in the presence of Fe^3+^ with either two **(A)** or three **(B)** hydroxamate groups close to the Fe^3+^ ion (FE is shown as a brown sphere). The insets show the corresponding systems in van der Waals sphere representations. The Na^+^ ion is light pink. The distances between FE and oxygen atoms are between 2 and 4 Å.

The flexibility of this trimer allows for such positioning of the hydroxamate oxygens that could coordinate iron(III) by either four or six oxygens (Figure 11). The other oxygen atoms that are attracted by Fe^3+^ in MD trajectories are from the amide terminal group or the peptide bond. However, these conformations result from classical MD trajectories, and for a detailed description of the final coordinated state quantum-mechanical approach would be necessary. This would require quantum-mechanical calculations to obtain force field parameters or quantum-mechanical molecular dynamics. However, we have shown that even with the classical molecular mechanics description of point charges, the distances of the iron(III) ion to these oxygens suggest that coordination of iron is possible and corroborates our CD experiments. The linear S_L_ peptide-like molecule is flexible enough to achieve a structure in which 6 oxygens are closely positioned to iron.

### 2.6 Molecular docking of the linear N^δ^-acetyl-N^δ^-hydroxy-l-ornithine trimer-Fe^3+^ complex and natural siderophore to the FhuD and FhuE proteins

Experiments with *E. coli* mutant strains indicate that the hydroxamate siderophore uptake pathway is involved in the internalization of the S_L_ siderophore mimic. To obtain further insight into molecular interactions of S_L_ with FhuD and FhuE, the key members of the hydroxamate siderophore uptake pathway, we performed molecular docking (see Materials and methods).

**Figure 12:**
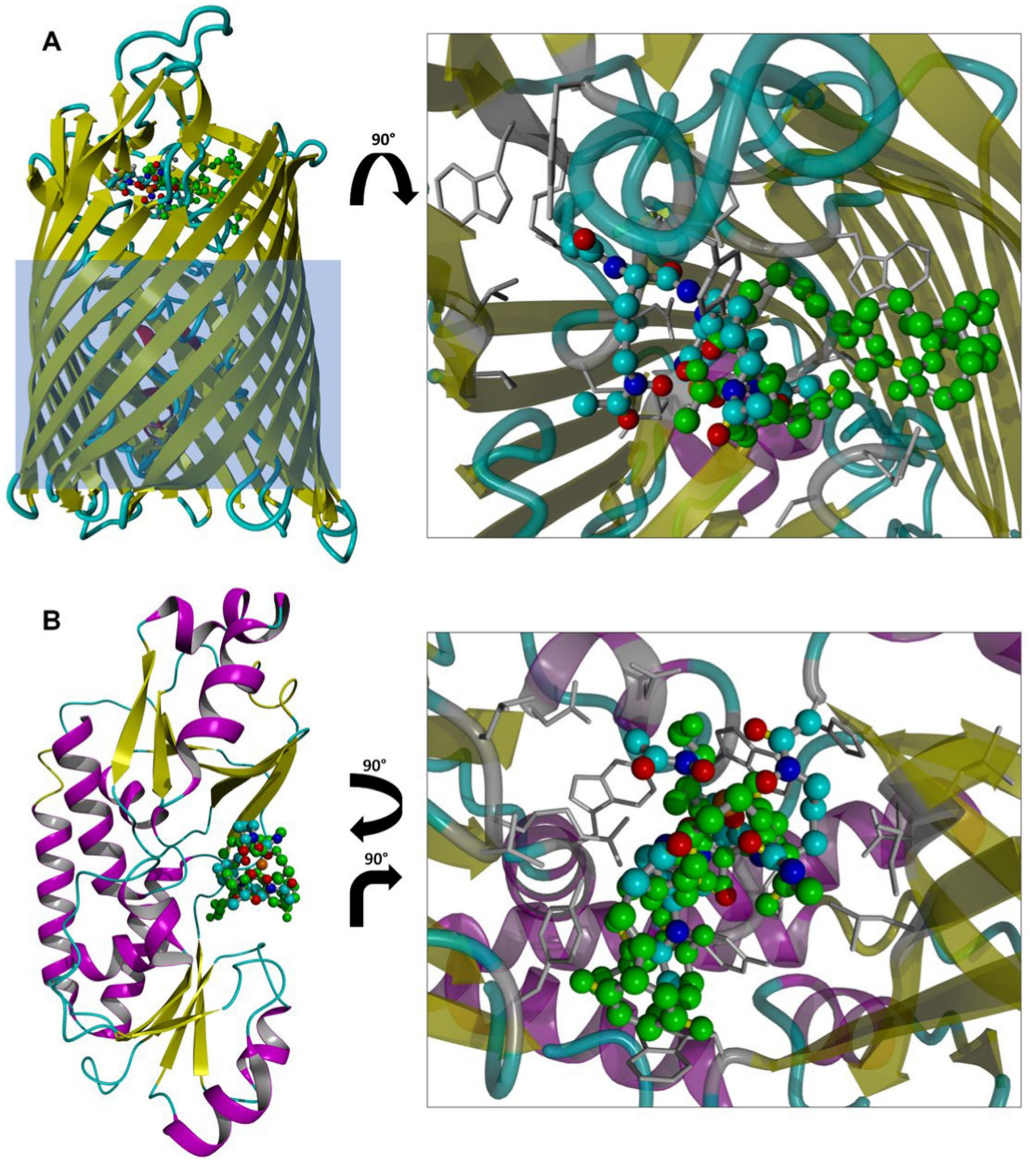
Top scoring poses of S_L_ docked to FhuE **(A)** or FhuD **(B)** structures. Structures from docking are superposed with the crystallographic ligand (coprogen; green ball-and-stick model). Protein residues contacting S_L_, defined as residues within 4 Å from the ligand, are shown as gray sticks. The light blue rectangle overlayed on FhuE depicts an approximate position of the *E. coli* outer membrane. Hydrogen atoms are omitted for clarity.

After validating the robustness of the docking protocol by successfully re-docking crystallographic ligands (with the ligand heavy atom root-mean-square deviations of ≤0.75 Å; Supplementary Figure S6), we generated docking poses of S_L_ bound to FhuD and FhuE. The resulting ligand poses largely coincide in terms of contacting amino acids with coprogen molecules present in the crystal structures of both proteins (Figure 12). They also possess favorable docking scores with predicted binding free energies of −9.3 and − 9.6 kcal/mol in FhuD and FhuE, respectively. These scores are by 2–3 kcal/mol worse than the scores assigned to the best poses of re-docked coprogen, i.e., −11.3 and − 12.6 kcal/mol for FhuD and FhuE, respectively. However, these estimates are consistent with coprogen being a much stronger natural ligand of hydroxamate siderophore uptake pathway proteins than S_L_. However, the S_L_ docking poses in FhuE show that the binding pocket is large suggesting that different binding modes for other siderophores may be possible and that FhuE could recognize and accommodate different ligand types.

## 3 Conclusion

Using modified ornithine we synthesized hydroxamate-type siderophore mimics and conjugated one of them with PNA oligomers. We found that the linear hydroxamate trimer coordinates iron(III), but its cyclic analog does not. CD spectroscopy showed that the ferric-S_L_ complex adopts the Λ configuration similar to ferrichrome. Molecular dynamics simulations confirmed that the S_L_ siderophore is internally flexible and showed that either four or six hydroxamate oxygens surround and interact with the iron(III) ion. In some cases, the S_L_ flexibility allows also the C-terminal amide and peptide bond oxygen to come at distances below 4 Å to the iron(III) ion.

The growth recovery assays on various *E. coli* mutants indicated that the uptake of the linear siderophore mimic occurs via the *E. coli* receptors recognizing the hydroxamate-type siderophores, and pointed to the FhuE outer-membrane receptor and FhuD periplasmic binding protein as necessary receptors. Molecular docking suggested that S_L_ is a plausible binder to FhuE and FhuD, similar to the natural coprogen siderophore, transported via the same pathway. The experiment with the S_L_-PNA_anti-*acpP*_ conjugate, targeting the essential *acpP* gene, suggested that two contradictory effects are present; the growth inhibition of the PNA_anti-*acpP*_ is probably countered by the growth promotion coming from S_L_. To overcome this obstacle another assay, based on RFP fluorescence with PNA_anti-*rfp*_ targeting the non-essential RFP gene carried on a plasmid, was introduced. These flow cytometry experiments showed that the conjugates of S_L_-PNA_anti-*rfp*_ entered *E. coli* cells. They suggest that the whole conjugate is recognized by the *E. coli* receptors and S_L_ transports PNA inside the cell. Therefore, the conjugation of PNA_anti-*rfp*_ with S_L_ via a non-cleavable triazole ring resulted in PNA silencing activity confirming that PNA reached the mRNA target in the cytoplasm. This study is a proof-of-concept that the Trojan horse strategy using the siderophore uptake pathway is possible for PNA, but to avoid the simultaneous growth promotion from the siderophore it needs to be optimized not to function as an iron(II) provider to bacteria.

## 4 Materials and methods

### 4.1 Reagents and conditions

Commercial reagents and solvents were used as received from the supplier. The Fmoc/Bhoc-protected PNA monomers (Fmoc-PNA-A(Bhoc)-OH, Fmoc-PNA-C(Bhoc)-OH, Fmoc-G(Bhoc)-OH, Fmoc-PNA-T-OH) were obtained from Link Technologies Ltd. and ASM Research Chemicals GmbH. The NovaSyn TG Sieber resin for PNA synthesis and Fmoc-Asp(Wang resin LL)-ODmab resin for preparing head-to-tail cyclic peptides were obtained from Novabiochem. The rink-amide resin (TentaGel S RAM) for peptide synthesis was obtained from Merck. The Nα-Fmoc-protected hydroxamate-ornithine derivative (Fmoc-l-Orn(Ac,OBz)-OH) was obtained from Iris Biotech GmbH, and Nα-Fmoc protected l-amino acids were obtained from Novabiochem (Fmoc-Gly-OH, Fmoc-azidolysine) and Merck (Fmoc-Lys(Boc)-OH). 10-Undecynoic acid and 6-azido-hexanoic acid were obtained from Merck and Novabiochem, respectively. The iron-free ferrichrome (desferrichrome) and deferoxamine mesylate salt were purchased from Merck.

Conjugation reactions were monitored using reverse-phase high-performance liquid chromatography (RP-HPLC). Analytical chromatography was performed using the Knauer C18 columns (5 μm particles, 4.6 × 250 mm) in buffer A (0.1% trifluoroacetic acid (TFA) in acetonitrile) and buffer B (0.1% TFA in water). The following conditions were used for HPLC: flow rate of 1.5 mL/min for 30 min, room temperature, UV/vis detection at 220 nm for siderophores and conjugates or 267 nm for PNA oligomers and conjugates. Additionally, the identity of the synthesized products was confirmed by mass spectrometry (MS) using the Q-TOF Premier mass spectrometer. All chemicals were of analytical or reagent grade, and the buffers were prepared using distilled water from a Direct-Q Millipore system.

#### 4.1.1 Synthesis of alkyne-PNA oligomers

All PNA oligomers were synthesized with the 10-undecynoic acid attached manually to the resin using Fmoc chemistry as previously described (Wojciechowska et al., 2014; Równicki et al., 2017; Castillo et al., 2018; see Table 3; Supplementary Figures S7–S10). PNA sequences are shown in Figure 6.

**Table 3:** Retention times (t_R_) and molecular masses of the synthesized compounds.

Compound name	Synthesized product	HPLC t_R_ [min]	HPLC method	Molecular mass [g/mol]		Yield^2^ [%]
				Calculated	Found^1^	
PNA_anti-*acpP*_	Alkyne – PNA_anti-*acpP*_	20.4	0–50%/ 30 min	2928.9	2929.5	6^a^
PNA_control_	Alkyne – PNA_control_	20.2	0–50%/ 30 min	2928.9	2929.3	7^a^
PNA_anti-*rfp*_	Alkyne – PNA_anti-*rfp*_	20.1	0–50%/ 30 min	4043.0	4044.0	7^a^
PNA_control2_	Alkyne – PNA_control2_	20.9	0–50%/ 30 min	4043.0	4043.5	6^a^
S_L_	Azido – (Orn(Ac,OH))_3_	19.3	0–50%/ 30 min	672.7	673.4	36^ab^
S_C_	cyc(Asp-(Orn(Ac,OH))_3_-Gly-Lys(N_3_))	13.5	15–30%/ 30 min	842.8	843.5	3^ab^
S_L_-PNA_anti-*acpP*_	(Orn(Ac,OH))_3_ – (CH_2_)_9_-PNA	18.5	0–50%/ 30 min	3601.6	3602.0	38^b^
S_L_-PNA_control_	(Orn(Ac,OH))_3_ – (CH_2_)_9_ – PNA_control_	18.1	0–50%/ 30 min	3601.6	3602.0	43^b^
S_L_-PNA_anti-*rfp*_	(Orn(Ac,OH))_3_ – (CH_2_)_9_-PNA_anti-*rfp*_	18.4	0–50%/ 30 min	4715.7	4716.5	18^b^
S_L_-PNA_control2_	(Orn(Ac,OH))_3_ – (CH_2_)_9_ – PNA_control2_	19.0	0–50%/ 30 min	4715.7	4716.0	29^b^

^1^Obtained from Q-TOF Premier mass spectrometer; ^2^estimated based on the ^a^resin loading or ^b^measuring the areas under the curves on the chromatograms. PNA oligomers include lysine at the C-terminus. For PNA sequences see Figure 6.

#### 4.1.2 Synthesis of linear hydroxamate siderophore: azido-(Orn(ac,OH))_3_

The azido-hydroxamate ornithine peptide was synthesized by manual SPPS. Standard Fmoc chemistry (Figure 2) was used with a 3-fold molar excess of the Fmoc-protected amino acids and rink-amide TentaGel S RAM resin (amine groups loading of 240 μmol/g; this resin has a linker which yields a C-terminal amide upon TFA cleavage of the peptide). Fmoc-protected modified ornithines (Fmoc-l-Orn(Ac,OBz)-OH) and 6-azido-hexanoic acid (N_3_-Hx-OH) were assembled as active derivatives with the use of O-(7-Aza-1H-benzotriazole-1-yl)-N,N,N′,N′-tetramethyluronium hexafluorophosphate (HATU) and with the addition of 1-hydroxy-7-azabenzotriazole (HOAt) and collidine (1:1:2) using the dimethylformamide/N-methylpyrrolidone (DMF/NMP; 1:1, v/v) solution for 60 min. Coupling efficiencies of amino acids were monitored using the Kaiser test (Wellings and Atherton, 1997). If the coupling reaction was not complete, the reaction was repeated with 1.5-fold molar excess of the amino acid for 45 min. The Fmoc deprotection was accomplished using 20% piperidine in DMF for 2 cycles (for 5 and 15 min). Benzoyl (Bz) protecting groups were removed from hydroxamate-ornithine derivative using 1 M solution of lithium hydroxide (LiOH) in methanol/tetrahydrofuran (MeOH/THF; 1:1; v/v) for 2 cycles (for 30 min each). Cleavage of the product from the resin was performed by treatment with a TFA/triisopropylsilane/water (95:2.5:2.5, v/v/v) mixture for 60 min. The obtained crude product S_L_ was lyophilized and subsequently analyzed by MS and purified by RP-HPLC (see Table 3; Supplementary Figure S11). The mobile phase gradients with buffers A and B were 0 to 50% for 30 min.

#### 4.1.3 Synthesis of cyclic hydroxamate siderophore: cyc(asp-(Orn(ac,OH))_3_-Gly-Lys(N_3_))

Fmoc-protected hydroxamate ornithine (Fmoc-l-Orn(Ac,OBz)-OH), glycine (Fmoc-Gly-OH) and azido-amino acid (N_3_-Lys(Fmoc)-OH) were attached to Fmoc-Asp(Wang resin LL)-ODmab resin (amine groups loading of 290 μmol/g; the acidic side-chain of the Asp is tethered to the resin; Figure 3). Manual SPPS was performed using Fmoc-deprotection and coupling reactions as described above. After completing the final Fmoc deprotection, the ODmab protective group was removed using 2% hydrazine in DMF for 3 cycles for 3 min each. With Asp acidic group deprotected the head-to-tail cyclization on resin was performed using N,N′-Diisopropylcarbodiimide (DIC) and HOAt (1:1), first for 2 cycles for 24 h each and later benzotriazolyloxy-tris[pyrrolidino]-phosphonium hexafluorophosphate (PyBOP), HOAt and N,N-Diisopropylethylamine (DIPEA; 1:1:2) for 4 h. Next, the protecting group (Bz) was removed and the product was cleaved from the resin as described above. The obtained crude product S_C_ was lyophilized and subsequently analyzed by MS and purified by RP-HPLC using mobile phase gradients with buffers A and B from 15 to 30% for 30 min (see Table 3; Supplementary Figure S12).

#### 4.1.4 Synthesis of PNA conjugates with hydroxamate siderophore

PNA-siderophore conjugates were synthesized using the CuAAC reaction (Figure 6). S_L_ (2.0 mg, 0.003 mmol) was dissolved in 0.1 mL of DMF/H_2_O (1:1 v/v) solution and 60 μL of the solution was added to 0.001 mmol of a PNA oligomer (2.9 mg of alkyne-PNA_anti-*acpP*_ or alkyne-PNA_control_, or 4.6 mg of alkyne-PNA_anti-*rfp*_ or alkyne-PNA_control2_) dissolved in 0.2 mL of DMF/H_2_O (1:1 v/v) solution. Copper(I) iodide (CuI; 1.0 mg, 5 μmol) and tris[(1-benzyl-1H-1,2,3-triazol-4-yl)methyl]amine (TBTA; 5.0 mg, 10 μmol) were dissolved in 0.5 mL of DMF/H_2_O (1:1 v/v) solution, stirred for 20 min and then added to the alkyne-PNA and azido-siderophore solutions. The reaction mixtures were stirred for 5 min before using ultrasounds for 30 min with the temperature of 50°C. The mixtures were centrifuged to remove the catalyst. The solutions, containing the crude products, were analyzed by MS and purified by analytical RP-HPLC. The mobile phase gradients with buffers A and B were 0 to 50% for 30 minutes unless stated otherwise (see Table 3; Supplementary Figures S13–S16). Siderophore-PNA solutions were prepared with distilled water. Concentrations of conjugates were determined by measuring the UV absorption at 260 nm (using Thermo Scientific Evolution 300 UV–Vis spectrophotometer) and calculated based on molar extinction coefficients provided by manufacturers.

### 4.2 CD measurements

CD spectra were recorded in aqueous buffer solution (100 mM phosphate buffer, pH 7.0), in the presence of 4 mM iron(III) salt solution (FeCl_3_ in the phosphate buffer). To obtain optimal recording conditions, siderophore concentrations were varied in the range 0.24–1.0 mM with an incubation time of 20–90 min (Supplementary Figure S1). In all CD experiments, the siderophore-Fe^3+^ molar ratio of 1:1 was maintained. The spectra were collected using the Biokine MOS-450/AF-CD spectrometer equipped with the Xe lamp using a 0.1 cm CD cell. The acquisition duration time was 2 s with a resolution of 1 nm. The measurements were performed in 100 mM phosphate buffer, pH 7.0, room temperature, and wavelength range 300–500 nm. The presented CD spectra were smoothed with the Savitzky–Golay method using GraphPad and are the averages of three scans. Each CD experiment was conducted twice to confirm the repeatability of the spectra.

### 4.3 General microbiology methods

If not stated otherwise, bacteria were cultured either in the lysogeny broth (LB) medium (VWR) or 50% Mueller Hinton Broth (MHB) medium (Difco). Bacterial stocks were prepared by mixing 1 mL of overnight LB culture of a given strain with 0.5 mL of autoclaved 50% glycerol (POCH). Liquid bacterial cultures were incubated in Innova 44 Incubator Shaker (New Brunswick Scientific) at 240 RPM. Optical density at 600 nm (OD_600_) of bacterial cultures was measured using SP-830 Plus Spectrophotometer (Metertech) with the clean medium as the reference. Wild-type *E. coli* K-12 was our in-house strain. All other *E. coli* K-12 mutants were acquired from the Keio collection (Table 2; Baba et al., 2006; Yamamoto et al., 2009).

#### 4.3.1 Preparation of *Escherichia coli* strains expressing the red fluorescent protein

Bacterial strains expressing RFP were prepared as described previously (Pieńko et al., 2021). Briefly, chemically competent cells were transformed with pBBR1MCS5(rfp) plasmid using the Kushner method. Following transformations bacteria were selected against gentamycin (20 μg/mL). The RFP expression was confirmed by measuring red fluorescence on the UV illuminator.

#### 4.3.2 Growth recovery assays

The growth recovery protocol was adapted from the literature (Zheng et al., 2012). 2 mL of LB medium was inoculated with a freezer stock of *E. coli* respective mutants and cultured overnight at 37°C with shaking. The overnight culture was then diluted 1:100 in 2 mL of fresh 50% MHB medium with or without the addition of 200 μM 2,2′-dipyridyl (DP, Merck). Bacteria were then cultured at 37°C with shaking until reaching OD_600_ = 0.6, and diluted to OD_600_ = 0.001. The diluted culture was aliquoted (90 μL per well) onto a 96-well, flat-bottom, fully-transparent plate. 10 μL of 10x concentrated solution of the siderophore that was previously either preloaded or not with iron (1:1 molar ratio siderophore: FeCl_3_) was added to each well. A series of Growth Controls (GCs) was prepared to allow compensation for additional iron added to the culture due to siderophore preloading. Plates were sealed with adhesive foil and incubated at 37°C with shaking for 19 h. Next, bacterial growth was measured as OD_600_ using the Tecan Sunrise plate reader. Each well condition was prepared in duplicate, and three independent biological replicates of each assay were conducted on different days. Data analysis included subtracting OD of Sterility Control (SC) and normalizing a given measurement to the appropriate GC.

#### 4.3.3 Flow cytometry

2 mL of LB medium was inoculated with a freezer stock of *E. coli* respective mutants and cultured overnight at 37°C with shaking. The overnight culture was then diluted 1:100 in 2 mL of fresh 50% MHB medium with 200 μM DP. Bacteria were then cultured at 37°C with shaking until reaching OD_600_ = 0.5, and diluted to OD_600_ = 0.001 in fresh 50% MHB medium with DP. 90 μL of such diluted culture was mixed with 10 μL of a tested compound dissolved in deionized water and cultured at 37°C with shaking for another 20 h. After that time bacteria were centrifuged at 5000 x g for 10 min and resuspended in 1 mL of FACSFlow buffer (BD Biosciences). Samples that required dead bacterial cells were then additionally incubated at 95°C for 5 min and cooled to room temperature. Next, 1 μL of SYTOX Red dead cell stain (Life Technologies) was added to each sample. After 30 min of incubation in darkness at room temperature samples were measured using LSRFortessa Cell Analyzer (BD Biosciences). For each sample, 10,000 events were recorded. Based on side-scatter and forward-scatter measurements, cells and within them single cells were identified. Within a single-cell population, only live cells were further analyzed. Gates for RFP-positive and RFP-negative cells were determined based on the measurements for non-treated bacteria expressing RFP and bacteria that do not express RFP, respectively (Supplementary Figures S17–S20). The experiment was repeated three times on different days.

#### 4.3.4 Dose-dependent RFP fluorescence silencing

Overnight cultures were prepared by inoculating 2 mL of 50% MHB medium with 200 μM DP with a freezer stock of *E. coli* and incubated at 37°C with shaking. Overnight cultures were then diluted 200 times of fresh 50% MHB medium with 200 μM DP and mixed with deionized water solutions of tested compounds (90 μL of bacteria +10 μL of the compound). Subsequently, bacteria were incubated for 3 h at 37°C with shaking, centrifuged, and resuspended in 500 μL of FACSflow buffer (BD Biosciences) containing SYTOX Red dead cell stain (Life Technologies; 1:1000 dilution). Cells were then analyzed as described in the Flow cytometry section.

### 4.4 Molecular dynamics simulations

Since S_L_ was found to both coordinate iron(III) and transport PNA inside *E. coli* cells, to understand the flexibility of this molecule and the dynamics of how iron is captured by the hydroxamate groups of S_L_, we performed molecular dynamics (MD) simulations. MD simulations were carried out for (Orn(Ac,OH))_3_ with the azide-linker at the N-terminus replaced with the COCH_3_ group for neutrality. The hydroxyls of the hydroxamate groups were either in protonated or deprotonated states, and the positions of the oxygens were either in *cis* or *trans* (Supplementary Figure S3). Various combinations were simulated because iron coordination occurs via deprotonated oxygens and in MD the barriers between the *cis* and *trans* conformation were too high to observe the transition. The free molecule and in the presence of iron(III) were simulated.

#### 4.4.1 Structure preparation and force field

The structures were drawn with *molview.org* and converted using *Openbabel*. The Amber ff14sb force field was used and the parameters for N^δ^-acetyl-N^δ^-hydfroxy-l-ornithine were derived using the AmberTools21 suite (Case et al., 2021). Atomic partial charges and force field atom types were assigned using *antechamber* with the *AM1-BCC* method for charge derivation (Jakalian et al., 2000), which was sufficient for our purposes. The Amber atom names, types, and partial charges are shown in Supplementary Figures S21–S22. The OPC3 (Izadi and Onufriev, 2016) parameters were used for water molecules, which were added to provide at least a 15 Å thick layer from the solute. In the case of simulations with ions, 11 Cl^−^ ions and 11 Na^+^ ions were included to ensure ionic strength of 150 mM NaCl. The systems formed a periodic cubic box with a size of 50.1 Å^3^ (Supplementary Figure S23). For the Na^+^, Cl^−^, and Fe^3+^ ions, the parameters of Li and Merz (12–6 normal usage set) were applied (Li et al., 2021; Sengupta et al., 2021).

The approaches to reproduce iron(III) binding included five series of simulations. Four water molecules at least 10 Å from the solute were randomly replaced with one Fe^3+^ ion and 3 Cl^−^ ions to achieve electroneutrality of the system. The starting conformations for the simulations including Fe^3+^ were taken as cluster representatives from the simulations of the free system. In each case, three systems were generated that only differed by the initial position of the Fe^3+^ ion.

#### 4.4.2 Simulation protocol and trajectory analysis

MD simulations were performed with Amber 20 version (Case et al., 2021) of *pmemd* and included the following steps. First, the energy minimization was performed with *sander* using 1,000 steps of steepest descent and 1,000 steps of conjugated gradient methods, with restraints of force constant equal to 5 kcal/mol on peptide heavy atoms. In the second thermalization phase, the temperature was gradually increased in 100 ps increments from 10 to 310 K. Equilibration at 310.15 K included 100 ps simulations in the NVT ensemble, followed by 200 ps in the NPT ensemble (constant pressure of 1 atm), with restraints relaxed to 1 kcal/mol. Next, 200 ps simulations were performed in the NPT ensemble without any restraints. The production simulations in the NPT ensemble lasted 1 μs each and were performed three times with different starting velocities.

Langevin thermostat was used for temperature control, with a collision frequency equal to 0.1, and Monte Carlo barostat was used for pressure control. The integration time step of 1 fs was used and the SHAKE algorithm was applied for the bonds involving hydrogens. A 12 Å cutoff was used for nonbonded interactions and Particle Mesh Ewald for electrostatic interactions.

Trajectories were analyzed with *cpptraj* from *AmberTools*. Systems were visualized using VMD 1.9.3 (Humphrey et al., 1996). Plots were drawn with gnuplot 5.2. To assess the stability of the solute in the production trajectories, root-mean-square deviation (RMSD), atomic root-mean-square fluctuation (RMSF), and radius of gyration (RoG) were calculated for the solute heavy atoms. The exemplary RMSD and RoG for the protonated S_L_ system without iron are shown in Supplementary Figure S24. Clustering was performed in *cpptraj* using the *k-means* algorithm for combined trajectories. Based on the Davies-Bouldin index (DBI) and the pseudo-F statistic (pSF) and, additionally, the percentage of variance explained, SSR/SST, the selected number of clusters was four (Shao et al., 2007).

### 4.5 Molecular docking

Molecular docking was carried out in the YASARA Structure (Ozvoldik et al., 2021). The following PDB structures were chosen as receptors: 1esz (Clarke et al., 2002) and 6e4v (Grinter and Lithgow, 2019) for FhuD and FhuE, respectively. Crystal water molecules were deleted. Hydrogens were added assuming a pH equal to 7.4, and the hydrogen bond network was optimized using the OptHyd command (Krieger et al., 2012). The binding site in each structure was chosen by placing a cubic simulation cell around the crystallographic ligand. To thoroughly explore available protein cavities, the box length of 25 Å for FhuD and 30 Å for FhuE structure was applied. The crystallographic ligand (coprogen) was deleted from the receptor model before S_L_ docking.

Protonated coprogen molecules (PDB ligand IDs CPO and HWS for FhuD and FhuE structures, respectively; see Supplementary Figure S6) were used for validation of the docking protocol via re-docking. The representative starting conformations of the S_L_ molecule were taken from MD simulations described above, with Fe^3+^ well-coordinated by hydroxamate groups (Figure 11). For the ferric ligand to be handled properly by the docking protocol, dative metal coordination bonds preserving the distances between Fe^3+^ and hydroxamate oxygen atoms were created.

Initial docking was carried out using the AutoDock Vina protocol (Trott and Olson, 2010) via the YASARA dock_run macro. The number of docking runs was set to 25, the ligand remained flexible, and exhaustiveness was set to 32. Subsequently, the results were optimized using dock_rescore macro with all previously obtained docking poses rescored with an energy minimization followed by 12 local docking runs each using the Vina local scoring method. Side chains were free during minimization. Point charges and dihedral barriers were obtained from the YASARA2 force field (Krieger et al., 2009).

## Data availability statement

The raw data supporting the conclusions of this article are either in the Supplementary material or will be made available upon reasonable request.

## Author contributions

UT: Validation, Visualization, Writing – original draft, Writing – review & editing, Formal analysis, Investigation, Methodology. MB: Formal analysis, Investigation, Methodology, Validation, Visualization, Writing – original draft. MW: Investigation, Supervision, Writing – review & editing. JS: Investigation, Writing – review & editing, Formal analysis, Methodology, Validation, Visualization. PM: Investigation, Writing – review & editing, Visualization, Formal analysis, Methodology, Validation. JT: Visualization, Writing – review & editing, Conceptualization, Data curation, Funding acquisition, Project administration, Supervision, Validation, Writing – original draft.
