# Supplementary material for: Iron uptake pathway of Escherichia coli as an entry route for peptide nucleic acids conjugated with a siderophore mimic
Source: Front Microbiol. 2024 Jan 31;15:1331021. doi: 10.3389/fmicb.2024.1331021 (PMC10864483; doi:10.3389/fmicb.2024.1331021)
Supplement: Supplementary file 1 [file Data_Sheet_1.pdf]

Supplementary Material for

Iron uptake pathway of *Escherichia coli* as an entry route for peptide nucleic acids conjugated with a siderophore mimic

Uladzislava Tsylents[1], Michał Burmistrz[1], Monika Wojciechowska[1], Jan Stępień[1], Piotr Maj[1], Joanna Trylska[1*]

[1]Centre of New Technologies, University of Warsaw, Warsaw, Poland.

*Correspondence: joanna@cent.uw.edu.pl

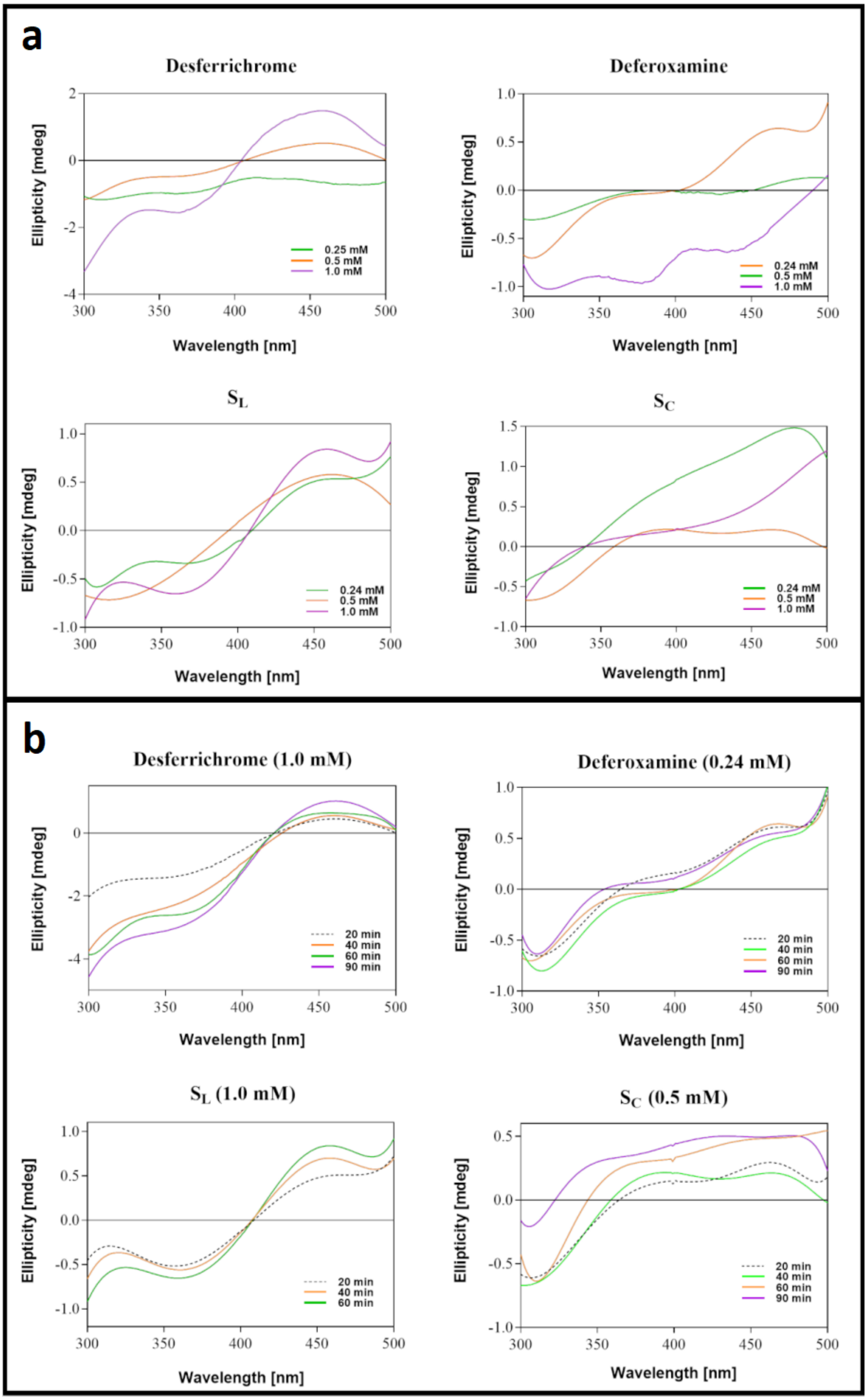

Supplementary Figure S1. CD spectra obtained for different concentrations (a) with various incubation time (b) of desferrichrome, deferoxamine and synthetic (S_L and S_C) siderophores mimics. In every CD run the molar ratio of Fe^{3+} to the siderophore was 1:1.

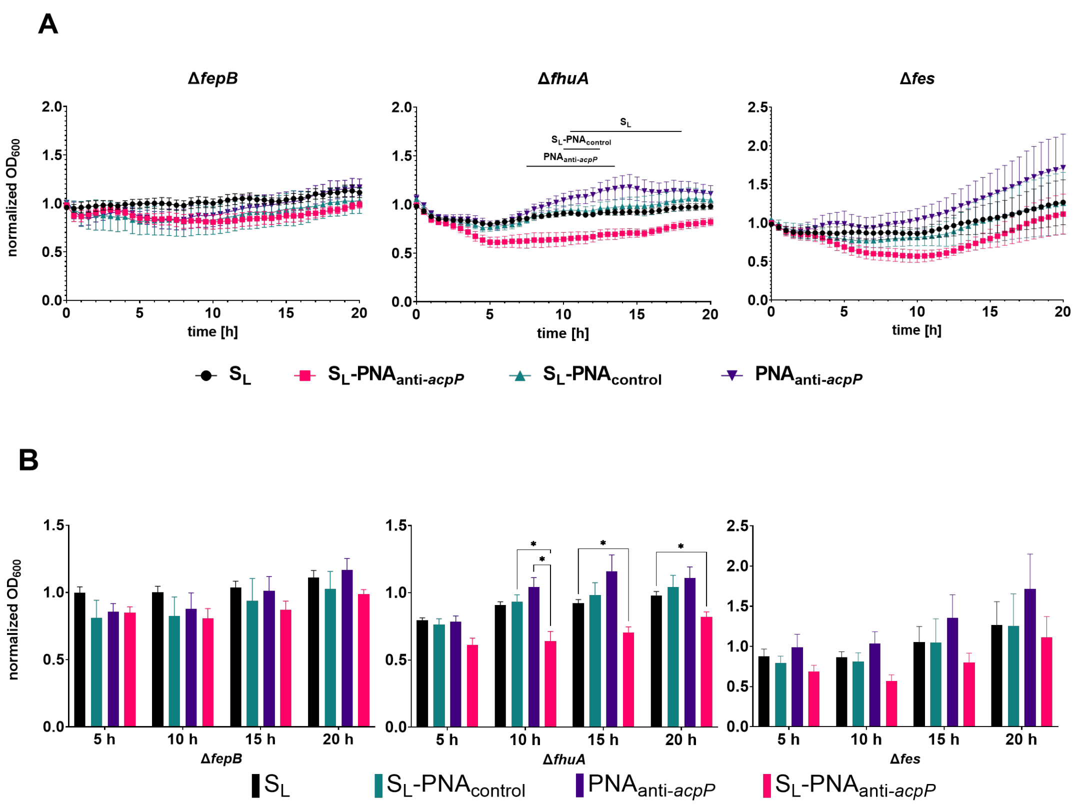

Supplementary Figure S2. A. Kinetic measurements for the growth recovery assay. Various *E. coli* K-12 mutants were cultured in iron-limiting conditions with or without compounds: S_L, its conjugate with $PNA_{anti\text{-}acpP}$ or $PNA_{control}$ (each at concentration of 16 µM). The growth of different strains was measured by OD_{600} and normalized to the OD_{600} measured for a given strain without the addition of any compound. The experiment was performed in two biological replicates, two technical replicates each. The errors shown are SEM, n=4. Statistical significance was tested by the two-way ANOVA test. Horizontal lines represent the time periods for which significant difference was observed between S_L-$PNA_{anti\text{-}acpP}$ and other compounds with P<0.05. **B.** Growth recovery assay as in **A** but for selected time points. Statistically significant differences referenced to normalized OD_{600} of S_L-$PNA_{anti\text{-}acpP}$ are marked with *, P<0.05).

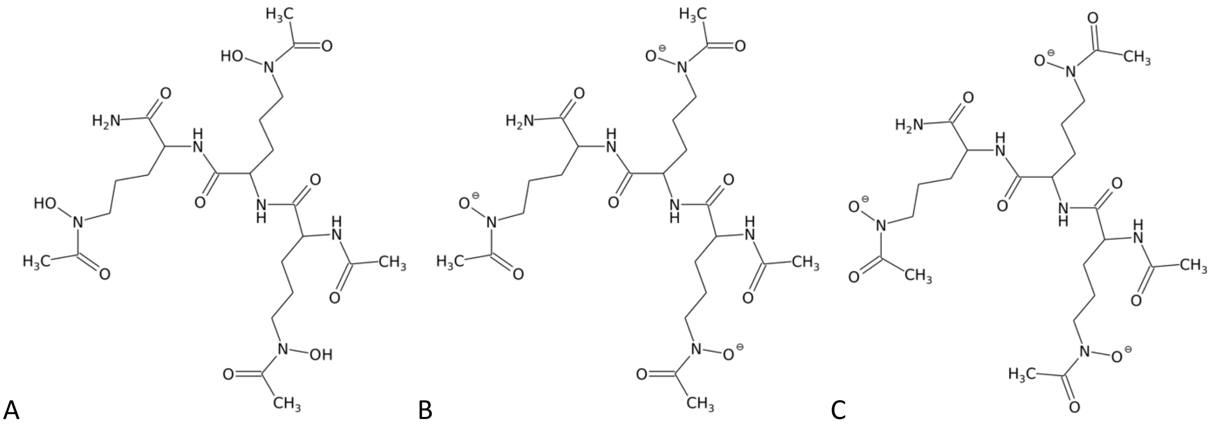

Supplementary Figure S3. Two-dimensional structures of the MD-simulated S_L siderophore variants with hydroxamate group oxygen protonated (A) and deprotonated in trans (B) and cis (C) positions. The C variant is optimal for iron(III) coordination.

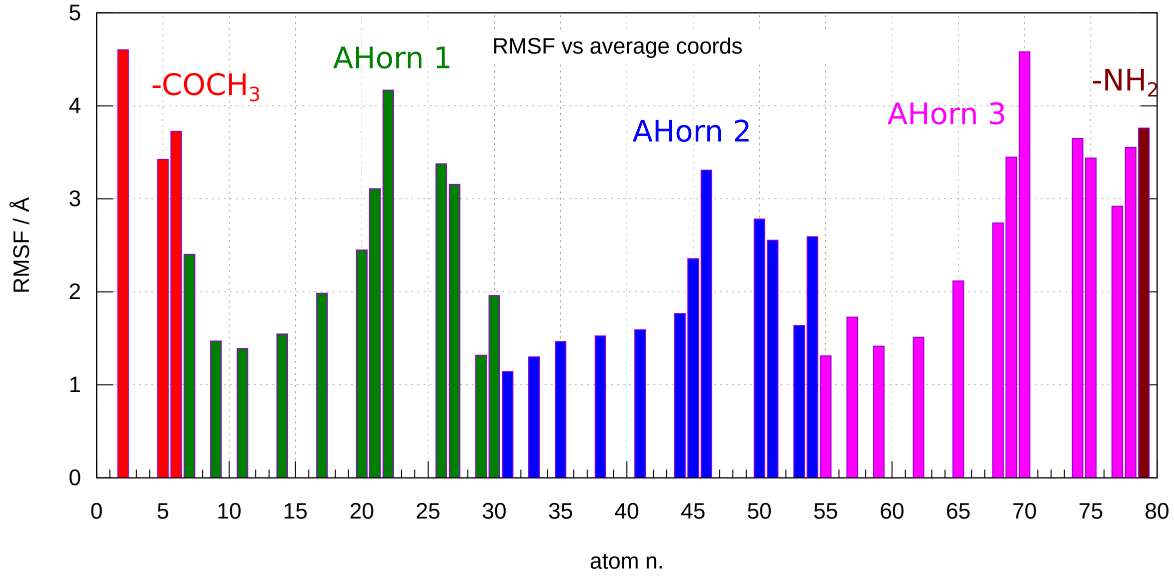

Supplementary Figure S4. The RMSF from the production simulation for the heavy atoms of the uncomplexed N$^{\delta}$-acetyl-N$^{\delta}$-hydroxyl-L-ornithine trimer (with its monomers labeled AHorn and shown in different colors). RMSF was calculated with respect to the average structure.

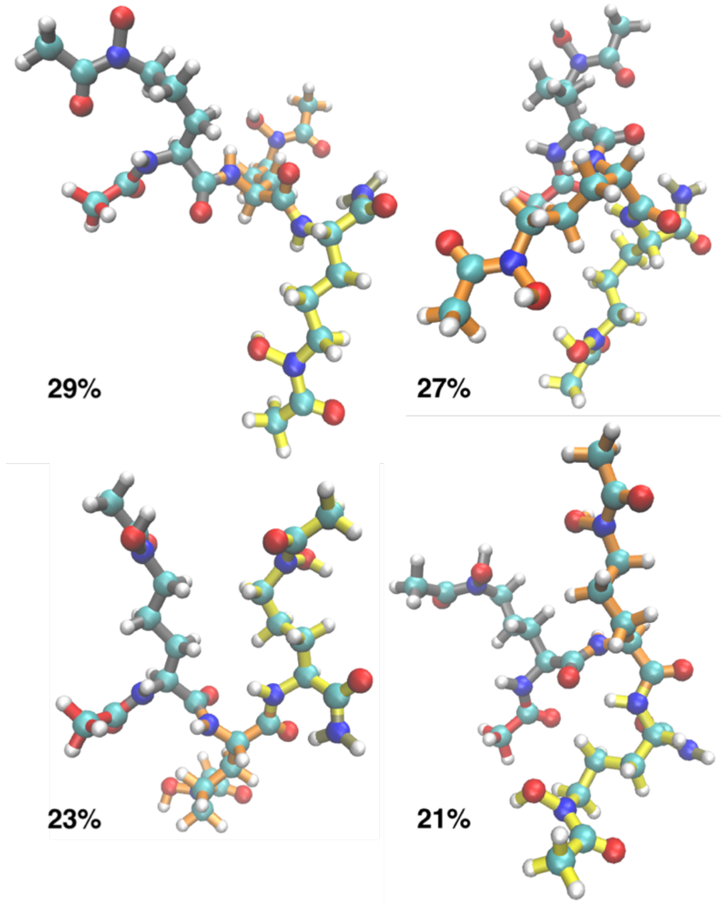

Supplementary Figure S5. Cluster representatives of the uncomplexed S_L siderophore derived from three MD simulations (based on 150000 frames) with the cluster occupancies shown in percent. The molecule is colored according to atom names and, in addition, to distinguish the residues in gray, yellow and orange.

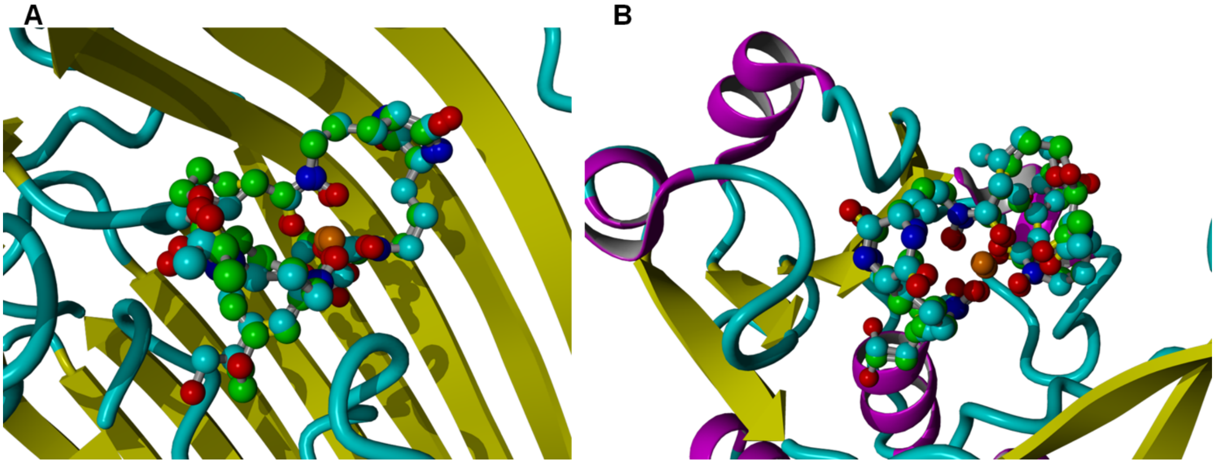

Supplementary Figure S6. Comparison of crystallographic (carbon atoms in green) and re-docked (carbon atoms in cyan) coprogen orientations within the binding sites of (A) FhuE (PDB ID: 6e4v) and (B) FhuD (PDB ID: 1esz) structures. Hydrogen atoms were omitted for clarity. Top-scoring docking poses were superimposed onto their respective crystal structures. The root-mean-square deviations between the crystallographic and re-docked poses calculated for the coprogen heavy atoms are 0.75 Å for FhuE and 0.56Å for FhuD. Of note, coprogen molecules deposited with the 6e4v and 1esz structures differ in the orientation around three asymmetric carbon atoms, hence their separate ligand ids, HWS and CPO, respectively. Ligands docked into their opposite structures, i.e., CPO docked into 6e4v and HWS docked into 1esz, resulted in slightly weaker docking scores but very similar poses to re-docked crystallographic ligands (data not shown).

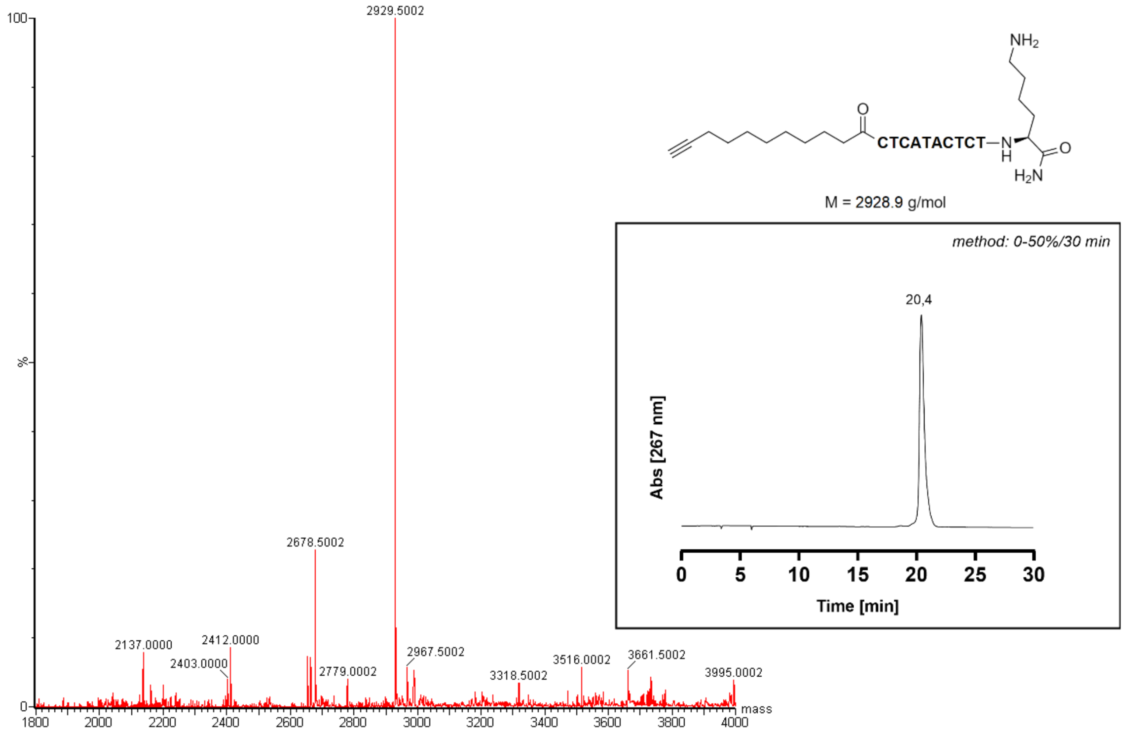

Supplementary Figure S7. The MS spectrum, HPLC chromatogram and the structure of the PNA$_{\text{anti-}\textit{acpP}}$ oligomer.

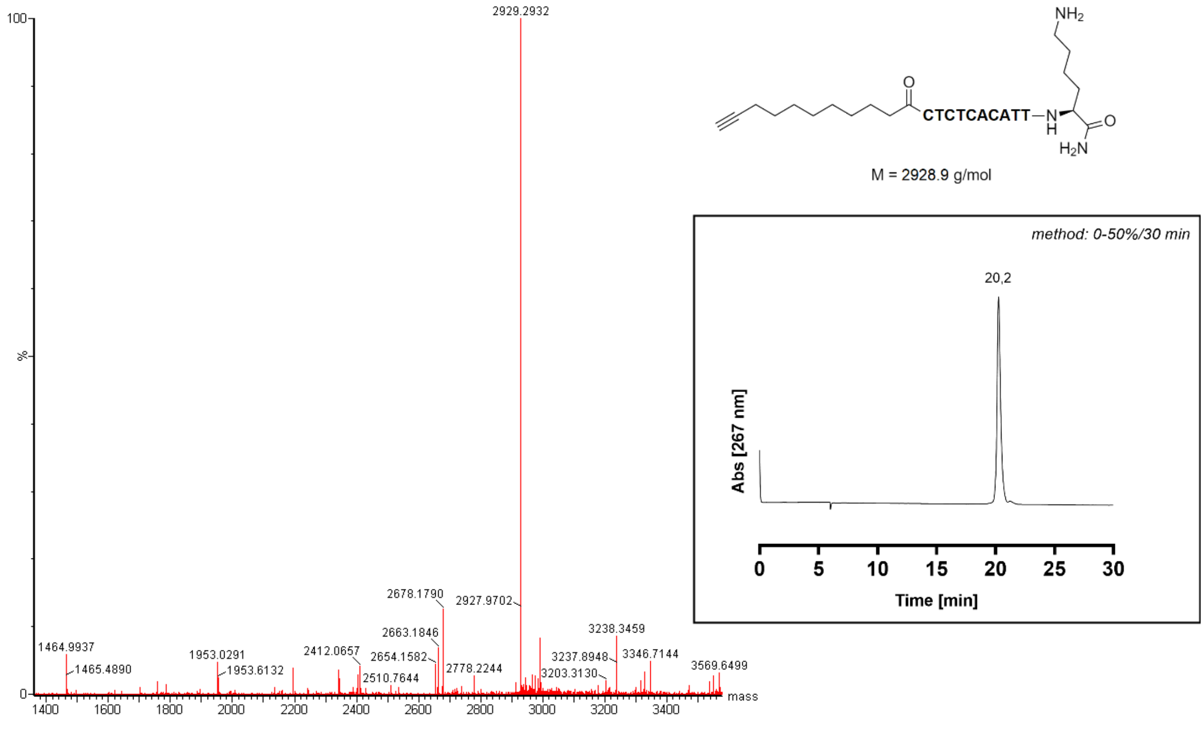

Supplementary Figure S8. The MS spectrum, HPLC chromatogram and the structure of the PNA$_{\text{control}}$ oligomer.

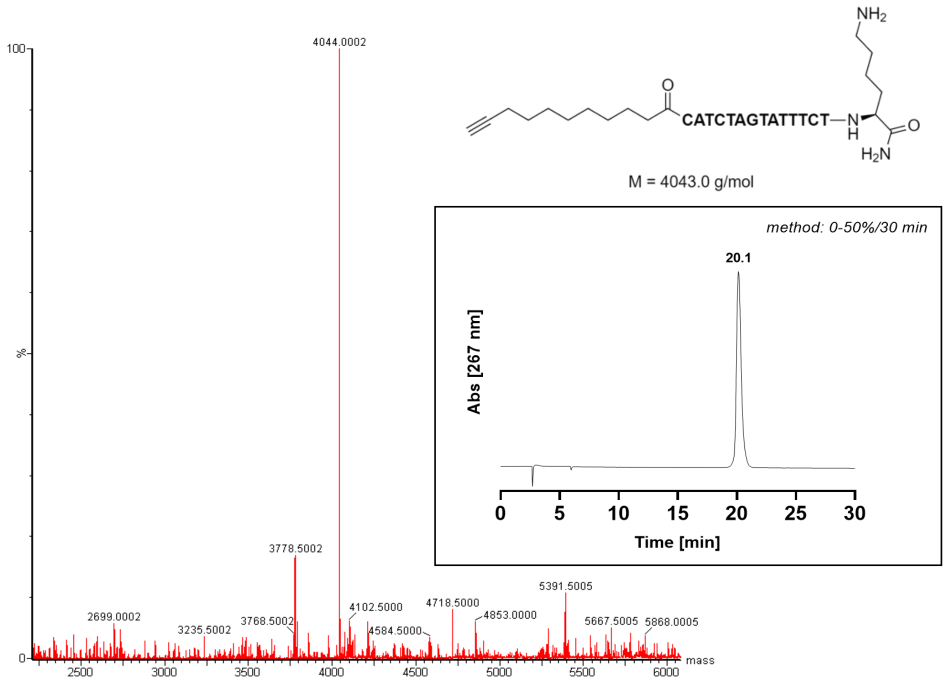

Supplementary Figure S9. The MS spectrum, HPLC chromatogram and the structure of the PNA$_{\text{anti-}\textit{rfp}}$ oligomer.

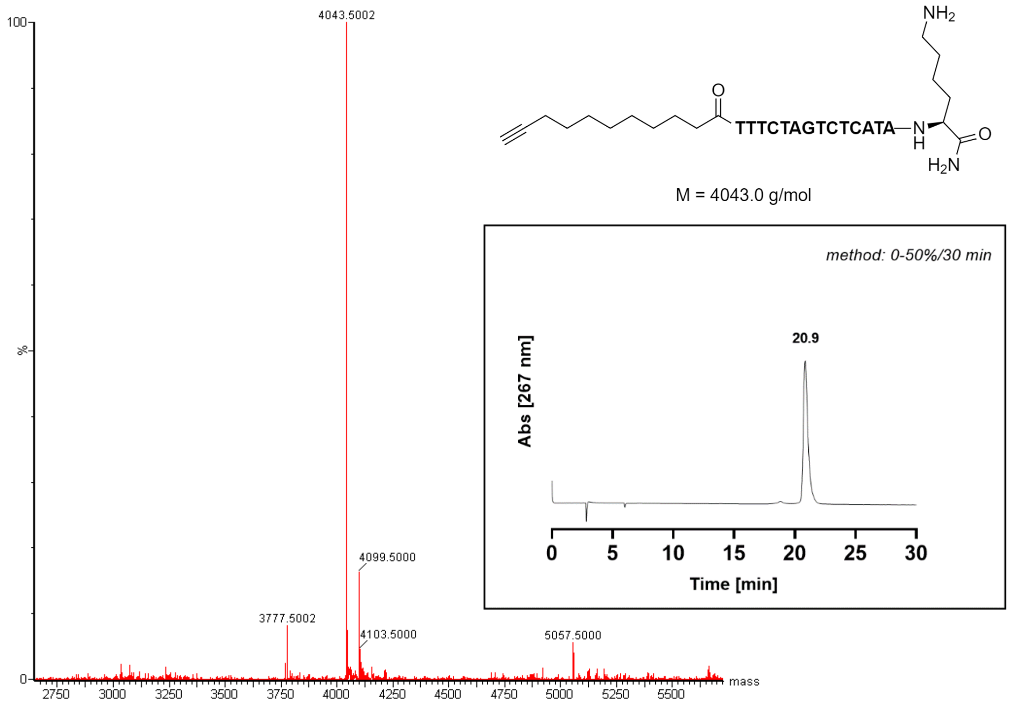

Supplementary Figure S10. The MS spectrum, HPLC chromatogram and the structure of the PNA$_{\text{control2}}$ oligomer.

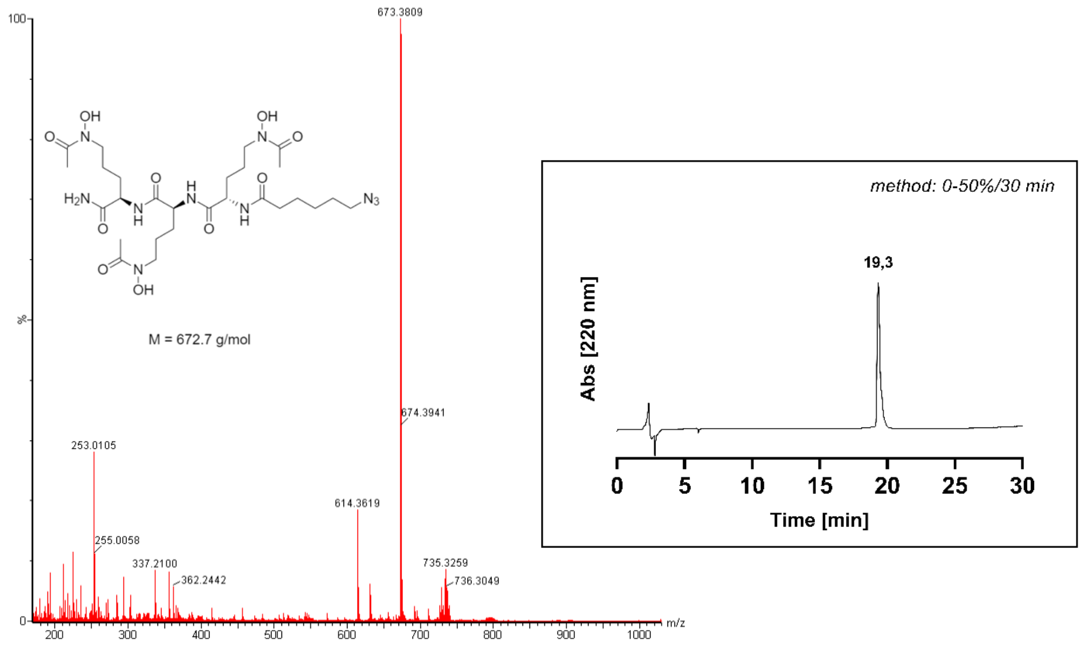

Supplementary Figure S11. The MS spectrum and HPLC chromatogram of the S_L siderophore with its structure.

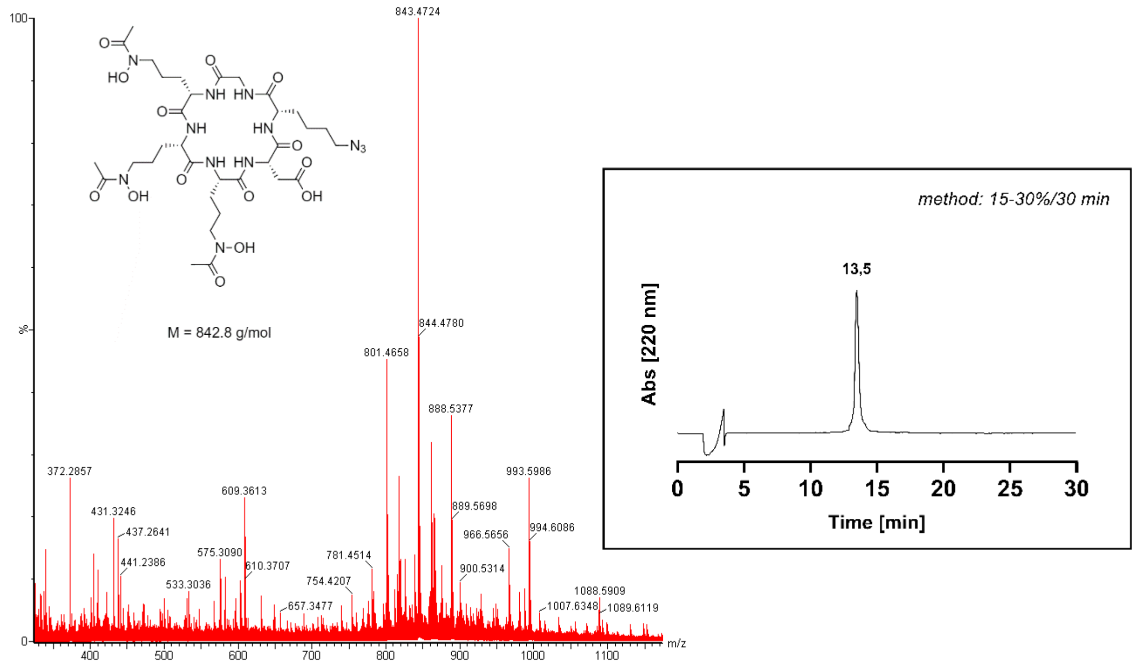

Supplementary Figure S12. The MS spectrum and HPLC chromatogram of the S_C siderophore with its structure.

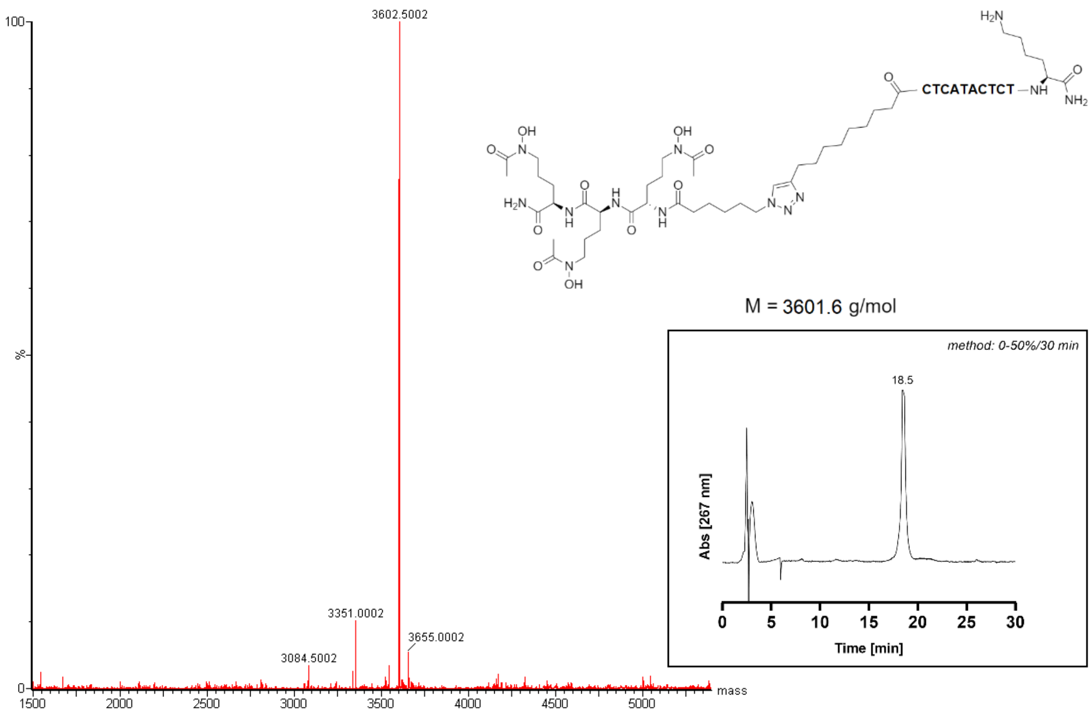

Supplementary Figure S13. The MS spectrum, HPLC chromatogram and the structure of the S_L – $PNA_{anti\text{-}\mathit{acpP}}$ conjugate.

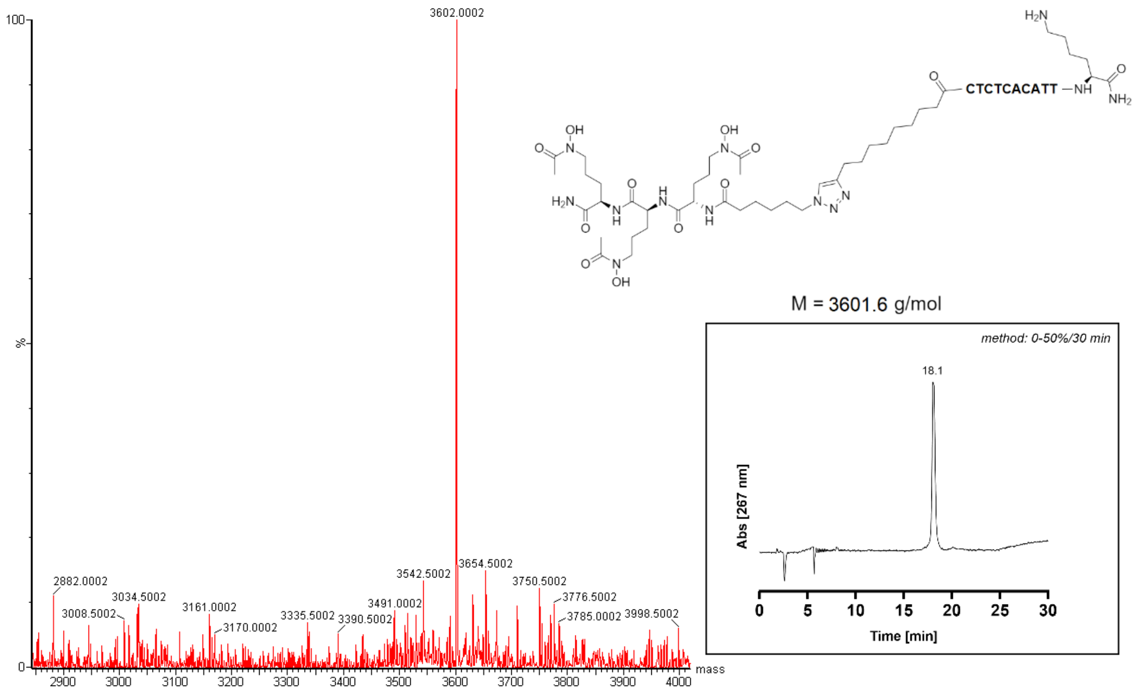

Supplementary Figure S14. The MS spectrum, HPLC chromatogram and the structure of the S_L – $PNA_{control}$ conjugate.

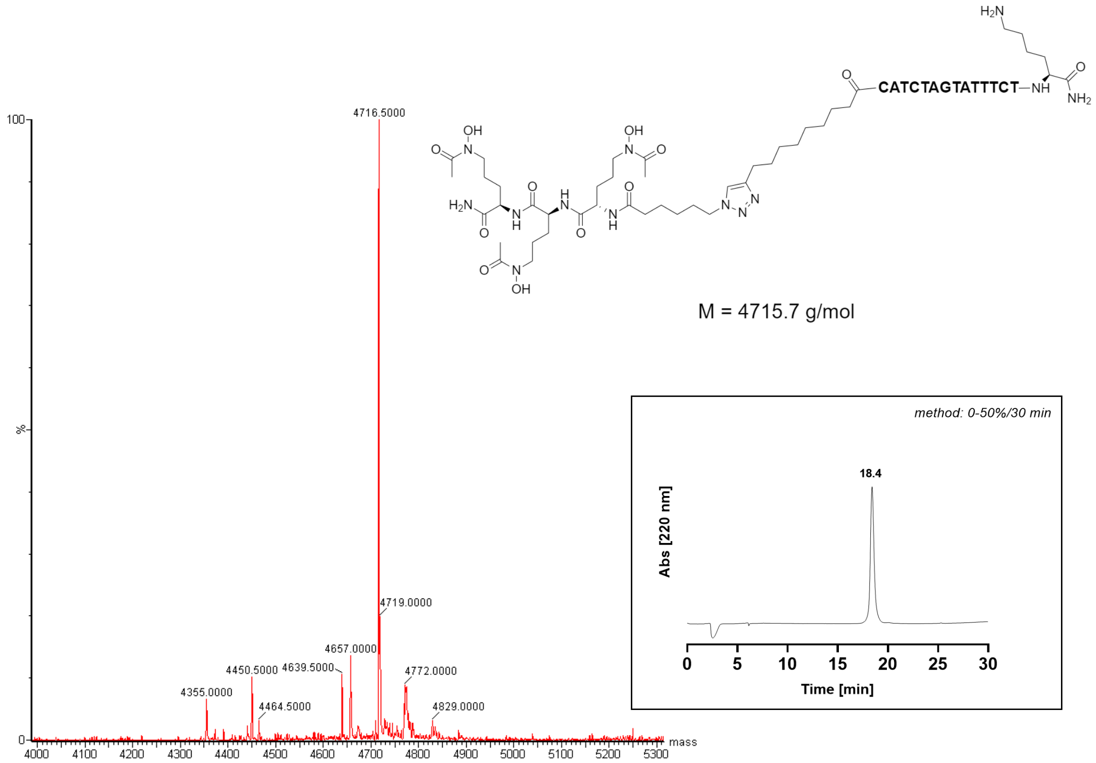

Supplementary Figure S15. The MS spectrum, HPLC chromatogram and the structure of the S_L – $PNA_{anti\text{-}rfp}$ conjugate.

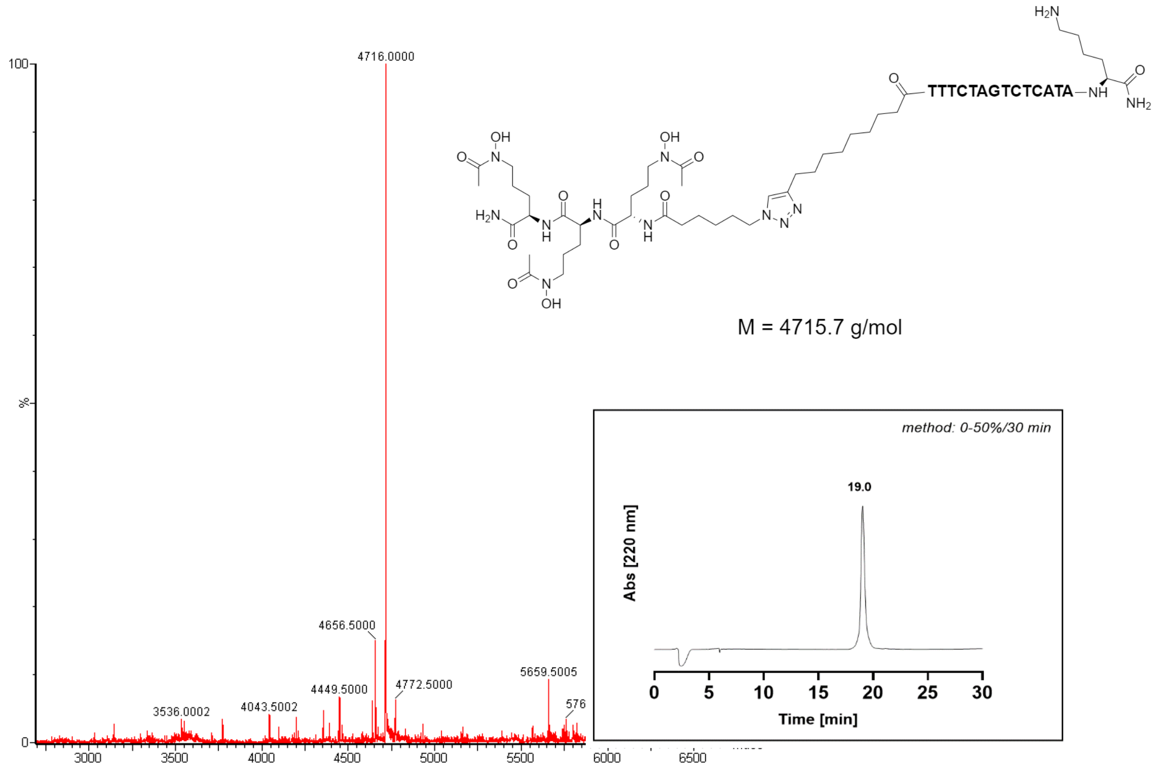

Supplementary Figure S16. The MS spectrum, HPLC chromatogram and the structure of the S_L – $PNA_{control2}$ conjugate.

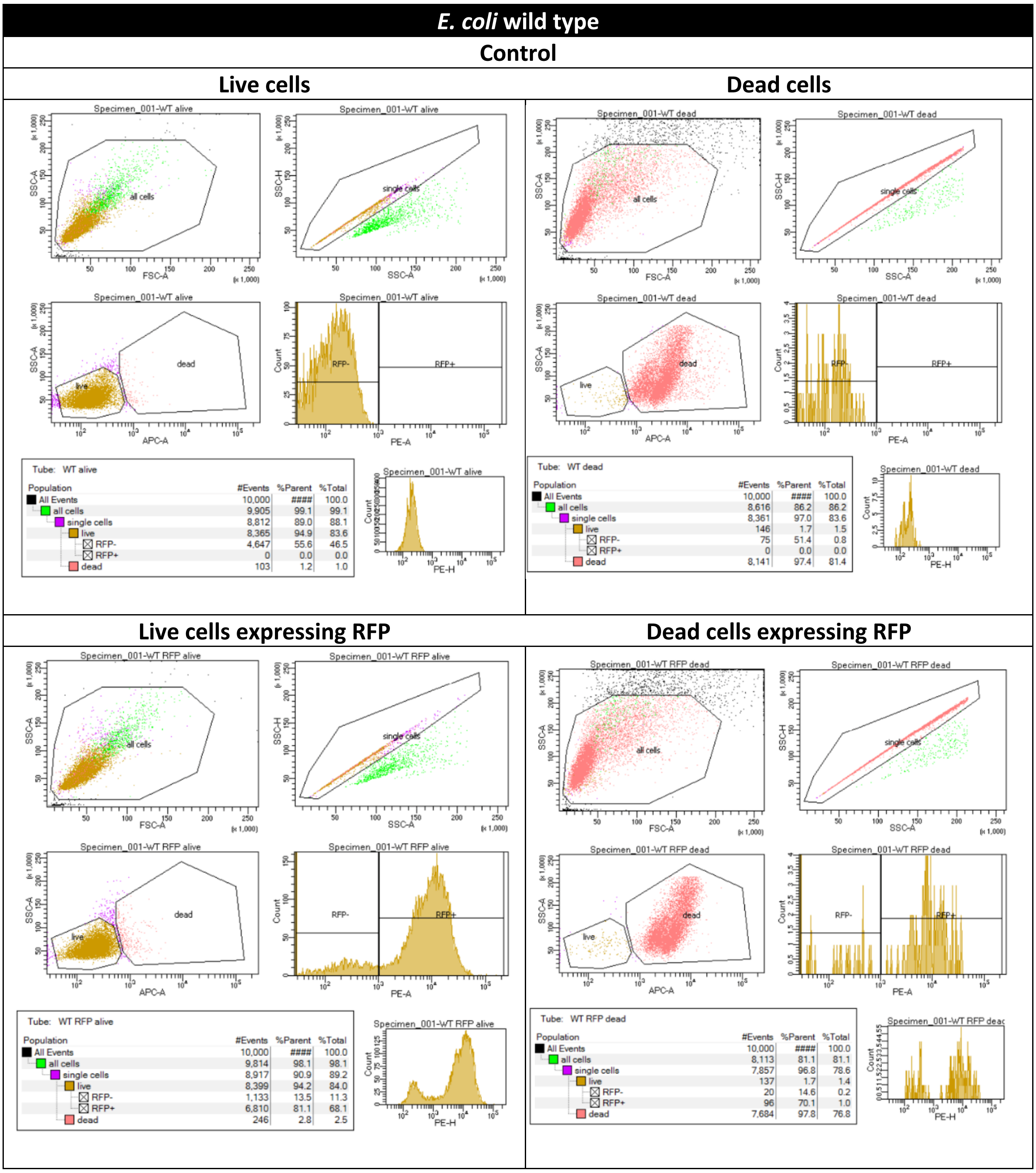

Supplementary Figure S17. RFP fluorescence detection for single cells of the *E. coli* wild-type strain with or without RFP expression using flow cytometry.

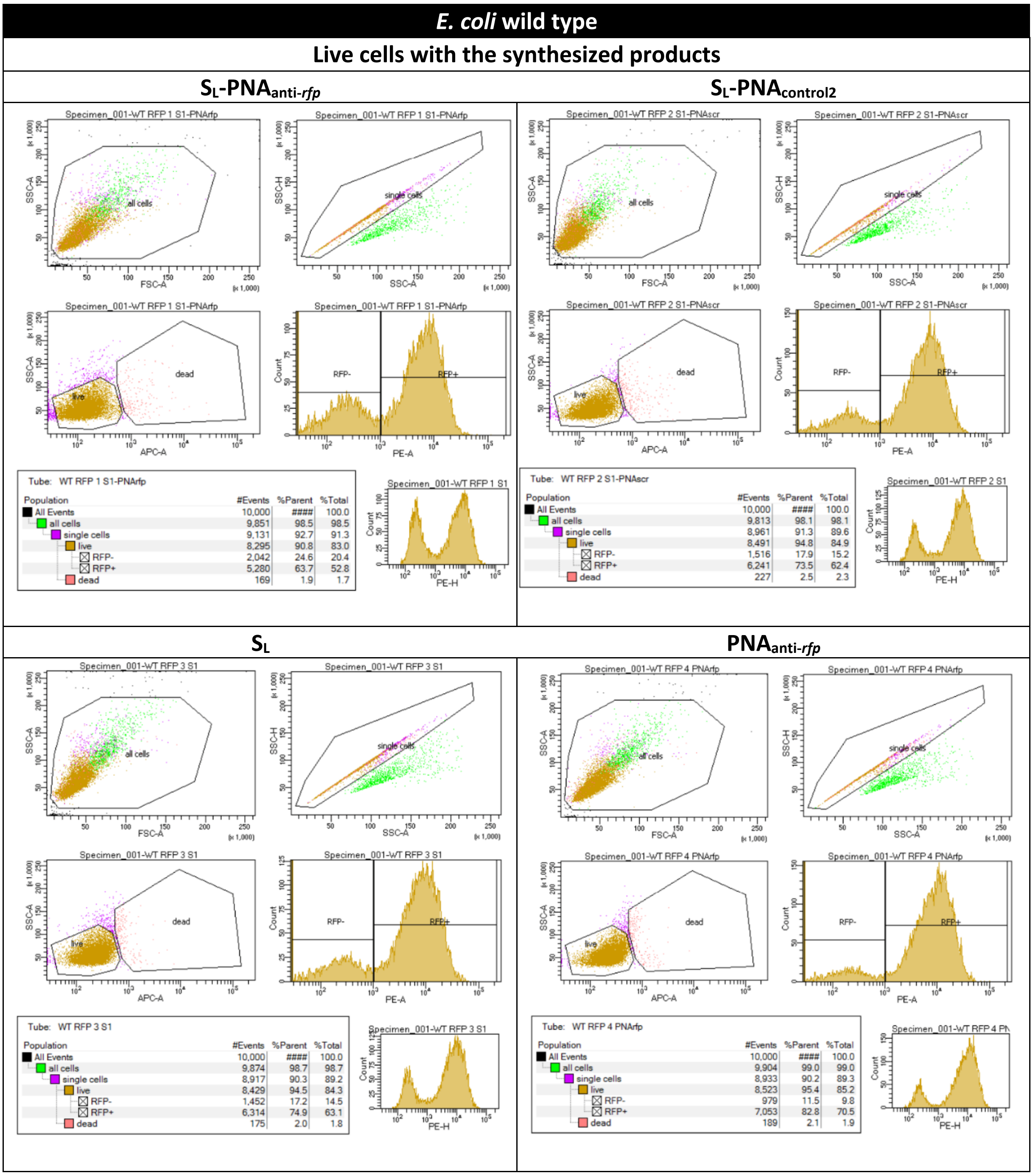

Tube: WT RFP 1 S1-PNArfp

Population	#Events	%Parent	%Total
All Events	10,000	####	100.0
all cells	9,851	98.5	98.5
single cells	9,131	92.7	91.3
live	8,295	90.8	83.0
RFP-	2,042	24.6	20.4
RFP+	5,280	63.7	52.8
dead	169	1.9	1.7

Tube: WT RFP 2 S1-PNAscr

Population	#Events	%Parent	%Total
All Events	10,000	####	100.0
all cells	9,813	98.1	98.1
single cells	8,961	91.3	89.6
live	8,491	94.8	84.9
RFP-	1,516	17.9	15.2
RFP+	6,241	73.5	62.4
dead	227	2.5	2.3

Tube: WT RFP 3 S1

Population	#Events	%Parent	%Total
All Events	10,000	####	100.0
all cells	9,874	98.7	98.7
single cells	8,917	90.3	89.2
live	8,429	94.5	84.3
RFP-	1,452	17.2	14.5
RFP+	6,314	74.9	63.1
dead	175	2.0	1.8

Tube: WT RFP 4 PNArfp

Population	#Events	%Parent	%Total
All Events	10,000	####	100.0
all cells	9,904	99.0	99.0
single cells	8,933	90.2	89.3
live	8,523	95.4	85.2
RFP-	979	11.5	9.8
RFP+	7,053	82.8	70.5
dead	189	2.1	1.9

Supplementary Figure S18. RFP fluorescence detection for *E. coli* wild-type strain in the presence of the synthesized products (at final concentration of 16 µM) using flow cytometry.

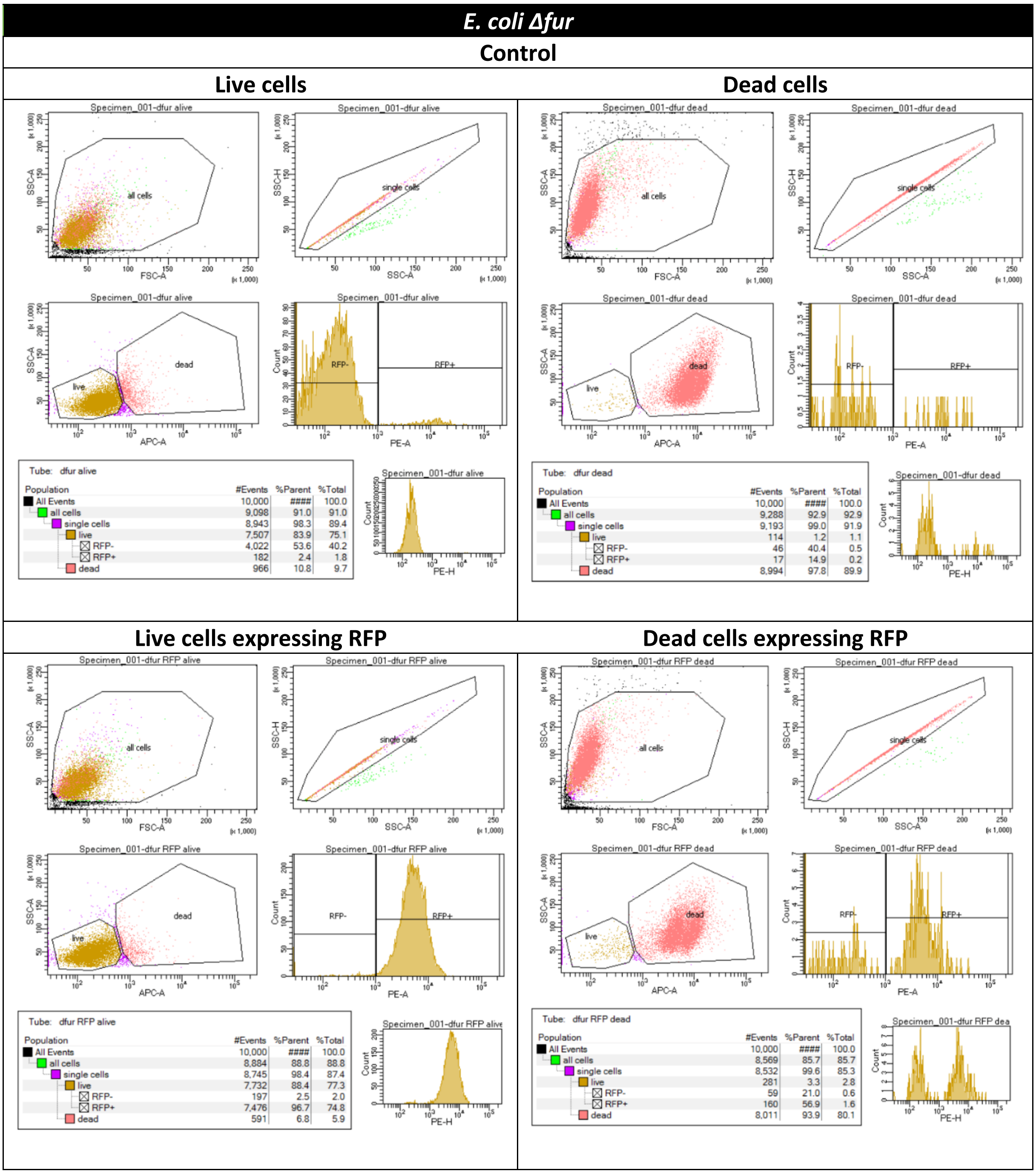

Supplementary Figure S19. RFP fluorescence detection for single cells of the *E. coli Δfur* strain with or without RFP expression using flow cytometry.

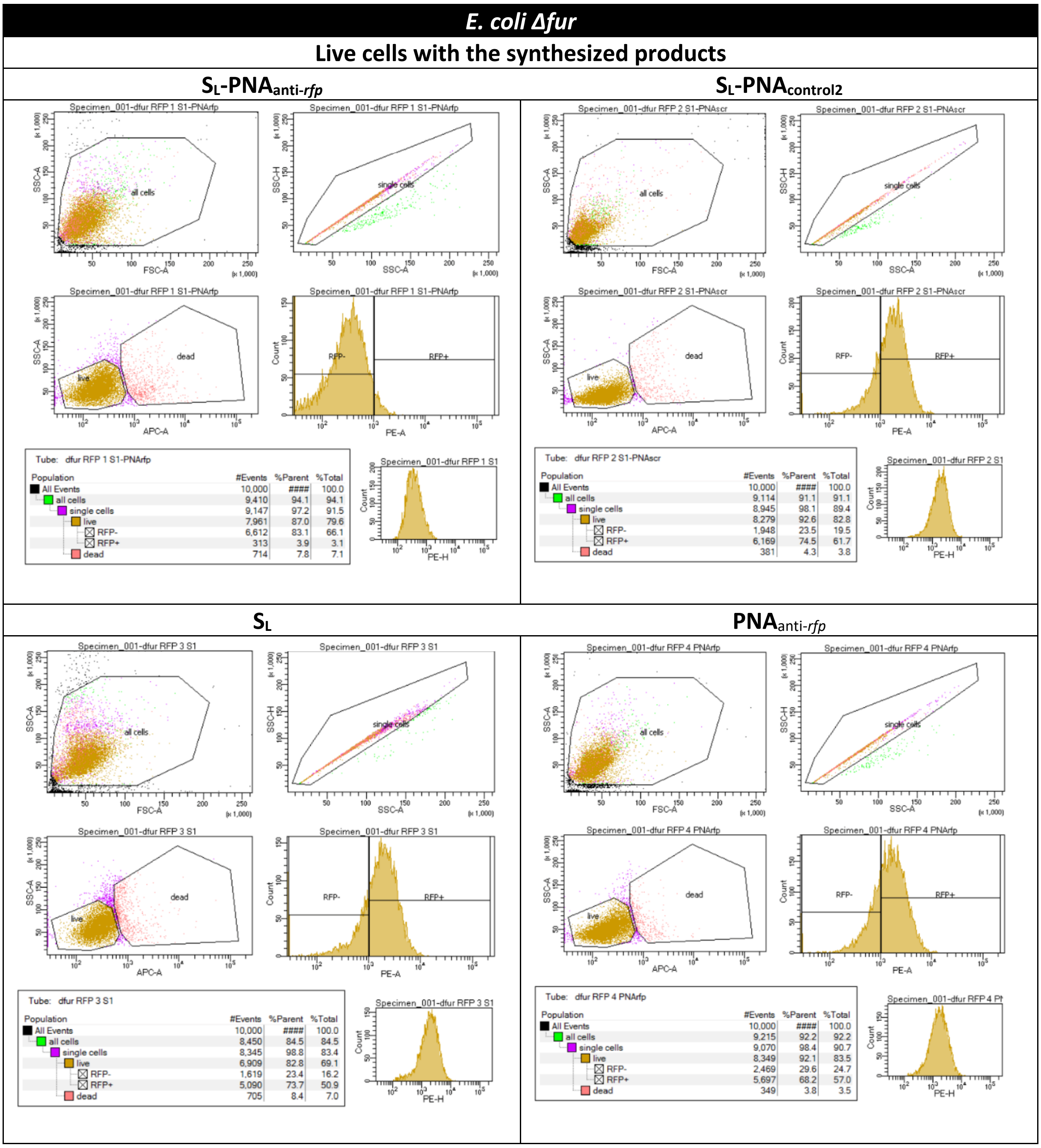

Supplementary Figure S20. RFP fluorescence detection for the *E. coli Δfur* strain in the presence of the synthesized products (at final concentration of 16 µM) using flow cytometry.

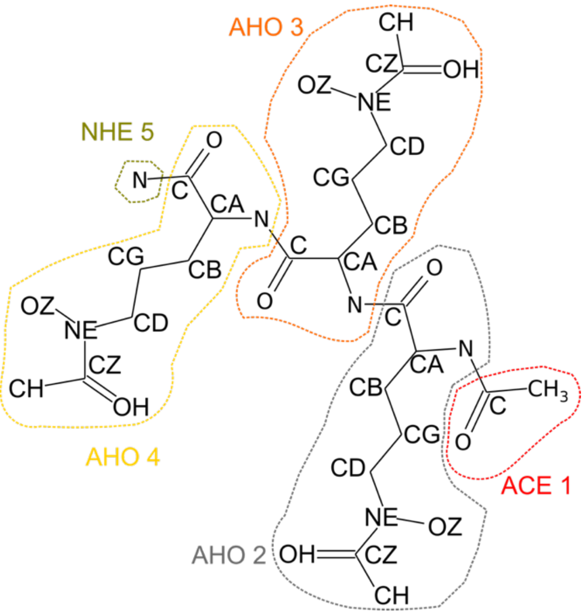

Supplementary Figure S21. Schematic structure of the simulated S_L molecule showing the residue and atom names used in the Amber files. For clarity hydrogen atoms are not shown. For deprotonated OZ systems, the residues are labelled AHD instead of AHO.

Atom		Partial charge [e]		
		Protonated	Deprotonated	
name	type		trans	cis
"N"	"N"	-0.522102	-0.547785	-0.502586
"H"	"H"	0.300209	0.304007	0.298840
"CA"	"CX"	0.040062	0.049620	0.027186
"HA"	"H1"	0.068316	0.067590	0.074023
"CB"	"CT"	-0.074713	-0.101564	-0.072065
"HB1"	"HC"	0.064280	0.056608	0.060786
"HB2"	"HC"	0.064280	0.056608	0.060786
"CG"	"CT"	-0.099486	-0.103567	-0.104464
"HG1"	"HC"	0.061757	0.045127	0.043986
"HG2"	"HC"	0.061757	0.045127	0.043986
"CD"	"CT"	0.050455	0.007987	-0.026509
"HD1"	"H1"	0.071848	0.031649	0.027186
"HD2"	"H1"	0.071848	0.031649	0.027186
"NE"	"N"	-0.286666	-0.052485	-0.047519
"CZ"	"C"	0.630792	0.523252	0.531599
"CH"	"CT"	-0.168551	-0.155351	-0.176823
"HH1"	"HC"	0.070671	0.034644	0.030579
"HH2"	"HC"	0.070671	0.034644	0.030579
"HH3"	"HC"	0.070671	0.034644	0.030579
"OH"	"O"	-0.548063	-0.722267	-0.624527
"OZ"	"OH"	-0.440056	---	---
"OZ"	"O2"	---	-0.693120	-0.667628
"HZ"	"HO"	0.421806	---	---
"C"	"C"	0.625747	0.649048	0.621200
"O"	"O"	-0.605535	-0.596063	-0.686381

Supplementary Figure S22. Atom names and types as well as atomic partial charges for different simulated S_L residue variants used in the simulations in Amber. For atom names in the S_L structure see Supplementary Figure S21.

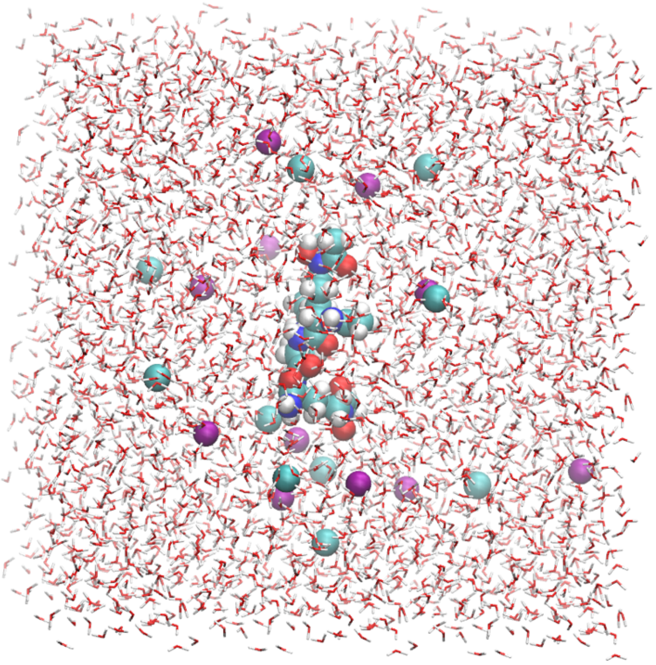

Supplementary Figure S23. The rectangular simulation box of various S_L variants showing the S_L molecule (colored by atom types) surrounded by the Na^+ (pink) and Cl^- ions (cyan) and water molecules.

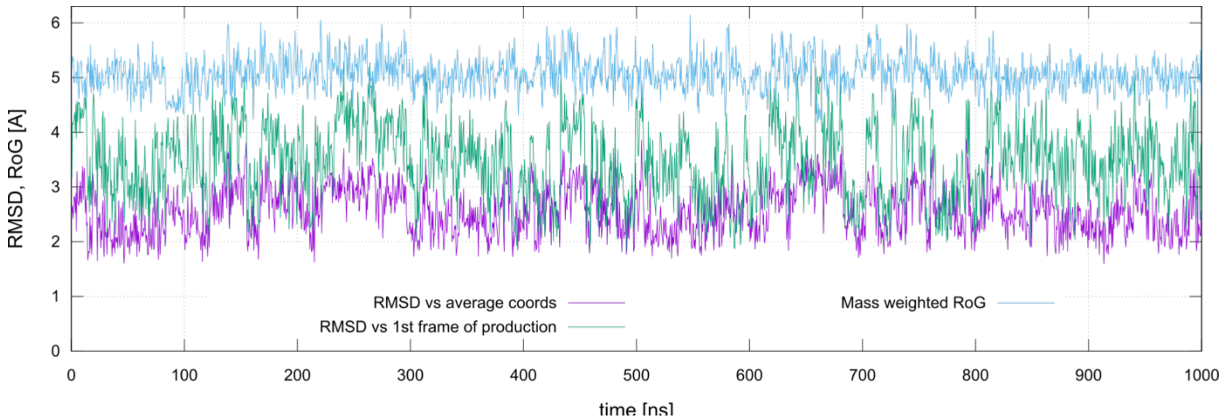

Supplementary Figure S24. Exemplary RMSD and RoG calculated based on one 1 µs long MD trajectory of the neutral S_L molecule (Figure S3A).
